# Innate immune cells in tumor immunity: dual roles, mechanistic insights, and therapeutic opportunities

**DOI:** 10.3389/fimmu.2026.1923822

**Published:** 2026-10-05

**Authors:** Songhang Xing, Jiayu Zhang, Shuang Wang, Yuan Hao, Dan Huang, Manwei Hao, Wurihan Bao, Xiaojie Qu, Yanyan Chi, Xuefeng Li

**Affiliations:** 1 Cancer Center, The First Hospital of Jilin University, Jilin University, Changchun, China; 2 Department of Breast Surgery, General Surgery Center, The First Hospital of Jilin University, Jilin University, Changchun, China; 3 School of Life Sciences, Jilin University, Changchun, China

**Keywords:** immune suppression, innate immune cells, therapeutic strategies, tumor immunity, Tumor microenvironment (TME)

## Abstract

The tumor microenvironment (TME) is a complex ecosystem that dynamically regulates tumor progression and therapeutic responses. Within this environment, innate immune cells play prominent dual roles, acting as both critical mediators of anti-tumor surveillance and facilitators of tumor immune escape. This review comprehensively synthesizes the phenotypic plasticity and functional heterogeneity of key innate immune cell subsets in tumor immunity, including tumor-associated macrophages (TAMs), natural killer (NK) cells, tumor-associated neutrophils (TANs), dendritic cells (DCs), as well as emerging players such as γδ T cells and innate lymphoid cells (ILCs). Mechanistically, we dissect core pathways governing innate immune cell function in the TME: the cGAS-STING pathway regulates type I interferon responses and immunogenicity; the NF-κB/STAT3 network coordinates inflammation and immunosuppression; the PD-1/PD-L1 axis mediates T cell exhaustion and innate cell dysfunction. Additionally, tumor-driven metabolic reprogramming and extracellular vesicles (EVs) intercellular communication collectively shape the immunosuppressive TME. We further evaluate emerging therapeutic strategies targeting innate immunity. This narrative review provides a comprehensive mechanistic framework and theoretical basis for developing precise, innate immunity-targeted cancer immunotherapies.

## 1 Introduction

Malignant tumors are one of the leading causes of death worldwide. Their underlying biological mechanisms driving tumor initiation and progression have long been a core research priority in biomedicine. Over the past century, cancer treatment strategies have undergone several paradigm shifts-from anatomically-based surgical resection, to radiotherapy and chemotherapy targeting cellular proliferation, and further to precision-targeted therapies aimed at molecular mutations (1). However, the most revolutionary breakthrough in recent years has arguably shifted from tumor cells themselves to the host immune system, known as immunotherapy. Modalities represented by immune checkpoint inhibitors (ICIs) and chimeric antigen receptor T-cell (CAR-T) therapy have achieved remarkable and durable clinical responses in melanoma, non-small cell lung cancer, and hematologic malignancies, with some patients even achieving clinical cures (2, 3). This milestone has not only rewritten clinical guidelines for cancer treatment but also profoundly reshaped our understanding of tumor biology: cancer is not merely a product of genomic instability, but rather a systemic disease involving dynamic interplay with the host immune system (4). Nevertheless, immunotherapy still faces significant challenges in clinical practice. Only 20-30% of patients with solid tumors respond to current ICI therapies, while the majority exhibit primary resistance or develop acquired resistance following treatment (5, 6). This clinical dilemma has compelled researchers to re-examine the interactions between tumors and the host immune system. These interactions are physically rooted and functionally centered in the TME (7).

The concept of the TME traces back to Stephen Paget’s “seed and soil” hypothesis, proposed in 1889 (8). While studying metastatic patterns of breast cancer, he astutely observed that the colonization and growth of tumor cells (the “seed”) depended on a specific organ environment (the “soil”). Modern advances in cell biology and immunology have vastly extended this hypothesis. We now understand that tumor tissue is not merely an accumulation of malignantly proliferating cancer cells but rather a complex and highly heterogeneous ecosystem (9). Within this system, tumor cells intermingle with stromal cells (such as fibroblasts and vascular endothelial cells) and various infiltrating immune cells. These components interact through direct cell-cell contact, soluble factors, and EVs (10). Together, they construct a sanctuary that supports tumor growth, invasion, and immune evasion. Within this intricate ecological network, the composition, phenotype, and functional states of immune cells-collectively termed the tumor immune microenvironment (TIME). The TIME largely determines the tumor fate and the patient’s response to immunotherapy (11). Consequently, in-depth analysis of the components, regulatory networks, and dynamic evolution of TME has become a research frontier. This work is crucial for identifying next-generation immunotherapy targets (12).

The foundation for understanding the TME lies in the cancer immunosurveillance theory proposed by Burnet and Thomas and its subsequent refinement into the concept of cancer immunoediting by Schreiber and others. This theory divides tumor-immune interactions into three distinct phases: Elimination, Equilibrium, and Escape (13). During the Elimination phase, the innate and adaptive immune systems work in concert to recognize and eradicate malignantly transformed cells at the earliest stages of tumor formation. The microenvironment at this stage exhibits a prototypical anti-tumor immune state, representing an ideal therapeutic target. However, if some tumor cells survive through mechanisms such as downregulating antigen presentation or upregulating anti-apoptotic pathways, they enter a prolonged Equilibrium phase. In this phase, the immune system cannot completely eliminate the tumor, but it exerts continuous selective pressure to suppress its growth, maintaining the tumor in an occult, subclinical state (14). Eventually, tumor cells evolve more sophisticated immune evasion strategies through the accumulation of genomic instability and epigenetic modifications. They establish a microenvironment that actively suppresses anti-tumor immune responses by recruiting immunosuppressive cells and remodeling the metabolic landscape, thereby entering the Escape phase. This leads to the clinically apparent, explosive growth of the tumor (15).

The TME exhibits remarkable diversity and plasticity. It encompasses not only cytotoxic immune effectors such as CD8^+^ cytotoxic T lymphocytes (CTLs) and NK cells but is also infiltrated by a substantial number of immunosuppressive cell populations, including regulatory T cells (Tregs), myeloid-derived suppressor cells (MDSCs), and TAMs (16). These cellular components do not function in isolation within the microenvironment; instead, they form an intricate regulatory network. These cells possess features of both innate and adaptive immunity. They can respond rapidly in the early stages of tumorigenesis and modulate the polarization of the microenvironment through the secretion of specific cytokine profiles. Their dual roles in tumor immune surveillance and evasion are emerging as a new focal point of research (17–19).

Beyond the cellular determinants, the physicochemical and metabolic properties of the TME critically determine immune response outcomes. Tumor cells undergo significant metabolic reprogramming to support rapid proliferation. The Warburg effect exemplifies this most classically: tumors prefer glycolysis even under oxygen-sufficient conditions (20). This metabolic mode leads to severe glucose deprivation and substantial lactate accumulation within the microenvironment, resulting in an acidic milieu. This nutrient competition presents a formidable challenge for effector T cells, which also rely on glucose for clonal expansion and effector functions (21).

At the molecular communication level, EVs serve as crucial mediators beyond traditional cytokines and chemokines, and they play an indispensable role in shaping the TME (22). Tumor cell-derived exosomes carry parent cell-specific proteins, lipids, and nucleic acids (such as microRNAs and lncRNAs). They transmit oncogenic or immunosuppressive signals to surrounding immune or stromal cells through uptake by recipient cells. Elucidating the mechanisms of EVs mediated communication not only helps uncover the finely tuned regulatory network of tumor immune evasion but also provides a theoretical basis for the development of liquid biopsy and novel drug delivery systems.

The TME constitutes a dynamic network involving multiple cell types, signaling pathways, metabolic processes, and host-microbiome interactions. It serves both as a sanctuary where tumor cells survive and as the battleground where immunotherapies exert their effects. Although key nodes such as PD-1/PD-L1 and CTLA-4 have been identified and successfully translated into clinical treatments, our current interventional strategies remain inadequate when facing the substantial heterogeneity and adaptive evolution of the TME. Core challenges in contemporary tumor immunology include how to accurately map the spatiotemporal architecture of the TME, how to comprehend its intricate interaction networks from a systems biology perspective, and how to employ combinatorial strategies to transform “cold” tumors (immune-desert or immune-excluded phenotypes) into “hot” tumors (immune-inflamed phenotypes). This work constitutes a narrative, non-systematic review. Literature was selected based on the authors’ expert curation of high-impact, clinically and mechanistically relevant publications in the field, without formal systematic database searching or predefined inclusion/exclusion criteria.

## 2 Key innate immune cell subsets in tumor immunity

### 2.1 Tumor-associated macrophages

In the TME, Tumor-Associated Macrophages are often the most abundant infiltrating immune cell population, and their abundance is frequently correlated with poor patient prognosis (23). As integral components of the innate immune system, macrophages exhibit remarkable plasticity and heterogeneity, enabling them to adopt divergent roles across different stages of tumorigenesis and progression (24). Rather than existing in a single activation state, TAMs undergo dynamic phenotypic switching in response to microenvironmental cues, such as cytokines, metabolites, and hypoxia. This plasticity allows them to alternate between an anti-tumor phenotype that suppresses malignant growth and a pro-tumor phenotype that facilitates disease progression (25). This dual role not only highlights the complex mechanisms underlying tumor immune evasion but also establishes a crucial theoretical basis for targeted immunotherapy (26) (**Figure 1**).

**Figure 1:**
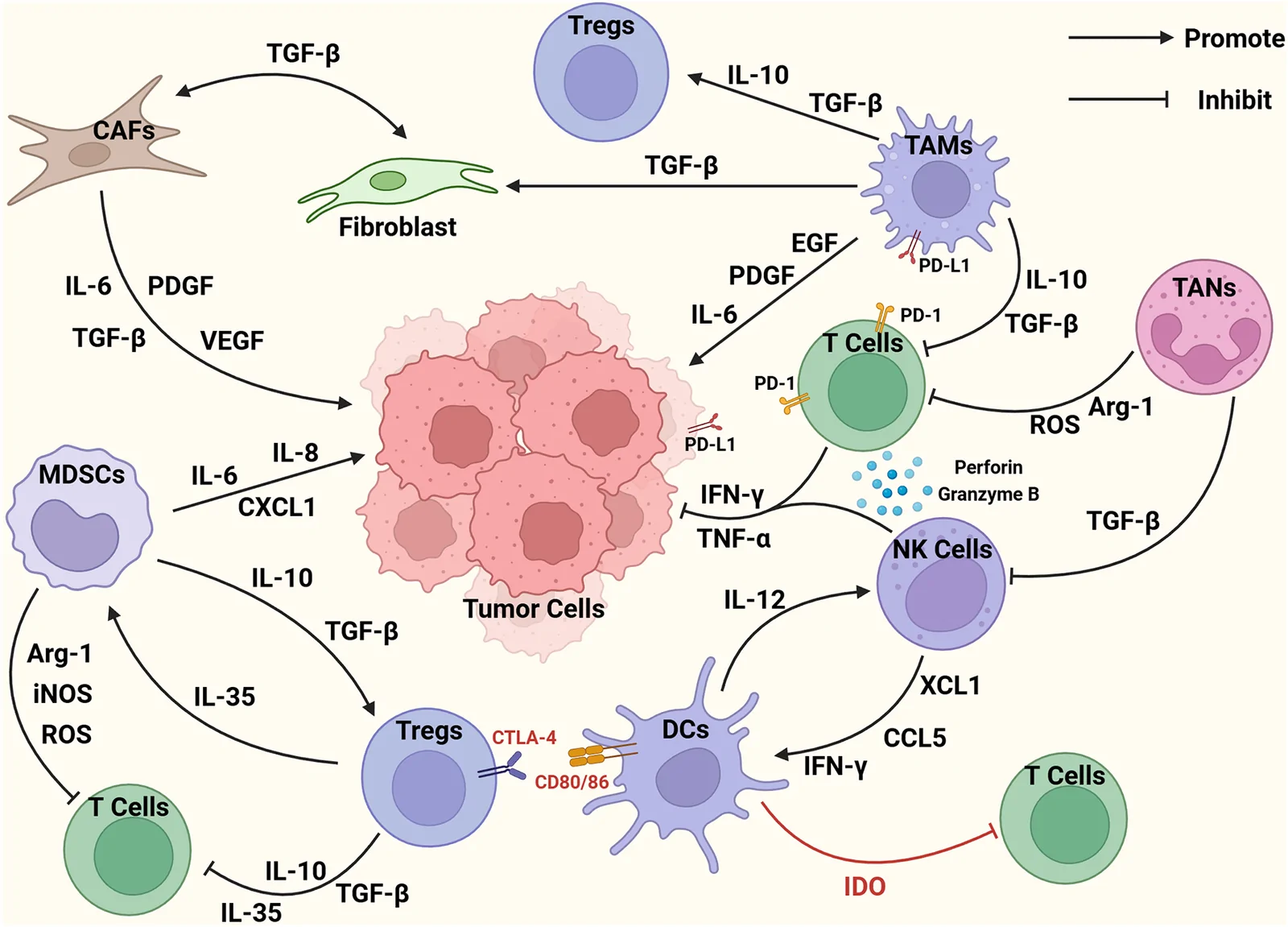
The complex network of cellular interactions and signaling pathways within the TME. This schematic illustrates the intricate crosstalk between tumor cells, stromal cells (CAFs and fibroblasts), and various immune populations (TAMs, TANs, MDSCs, Tregs, DCs, NK cells, and T cells). Tumor-promoting cells, such as CAFs and MDSCs, secrete pro-tumorigenic cytokines (e.g., IL-6, TGF-β, and VEGF) and inhibitory factors (e.g., Arg-1 and ROS) to suppress the cytotoxic activity of T cells and NK cells. The diagram further highlights key immune checkpoint pathways, such as PD-1/PD-L1 and CTLA-4/CD80/86, alongside the secretion of immunosuppressive cytokines like IL-10 and IL-35, and metabolic modulators like IDO, all of which collectively contribute to an exhausted immune landscape and facilitate tumor immune evasion.

Based on the classical dichotomous classification model, the polarization states of TAMs are generally conceptualized into classically activated M1 and alternatively activated M2 phenotypes (27). M1 macrophages are typically induced by pro-inflammatory signals such as interferon-γ (IFN-γ) and lipopolysaccharide (LPS), primarily exerting anti-tumor immune functions. They are characterized by high expression levels of MHC-II and co-stimulatory molecules (e.g., CD80 and CD86), the secretion of pro-inflammatory cytokines like IL-12 and TNF-α, and the upregulation of inducible nitric oxide synthase (iNOS). Through these effector molecules and efficient antigen presentation, M1 macrophages not only mediate direct cytotoxicity but also recruit NK cells and Th1 T cells, thereby participating in immune surveillance and clearance during the early stages of tumorigenesis (28).

However, as the tumor progresses, the TME frequently induces the transition of TAMs toward the M2 phenotype (29). M2 macrophages are primarily driven by IL-4, IL-10, or TGF-β, exhibiting immunosuppressive and tissue-repairing properties. Phenotypically, M2 TAMs highly express CD206, CD163, PD-L1, and arginase-1 (Arg-1) (30). Functionally, they mediate immune evasion by depleting microenvironmental L-arginine via Arg-1 and by secreting IL-10 and TGF-β to promote Treg generation, which consequently suppresses the activation and proliferation of effector T cells (29, 30). Furthermore, stimulated by the hypoxic microenvironment, M2 TAMs secrete pro-angiogenic factors such as vascular endothelial growth factor (VEGF) to facilitate tumor angiogenesis, invasion, and metastasis (**Table 1**).

**Table 1:** Core characteristics of innate immune cell subsets in the tumor microenvironment.

Major cell type	Functional subset	Functional phenotype	Specific surface & functional markers	Key regulatory signaling pathways	Applicable TME context	Clinical prognostic value
Tumor-associated macrophages (TAM)	M1-polarized macrophages	Anti-tumor	CD80, CD86, MHC-II, iNOS, CD11c	IFN-γ/JAK-STAT1, NF-κB, TLR pattern recognition signaling	Inflammatory TME, Th1 cytokine-enriched milieu, immunotherapy-responsive phase, early-stage tumors	Favorable prognosis; positively correlated with immune checkpoint blockade (ICB) response, enhances antigen presentation and tumor phagocytosis
	M2-polarized macrophages	Pro-tumor	CD163, CD206, Arg-1, IL-10, VEGF, CD11b	IL-4/IL-13/JAK-STAT6, IL-10/STAT3, TGF-β/Smad	Hypoxic TME, stroma-rich TME, immunosuppressive TME, intermediate/advanced-stage tumors	Unfavorable prognosis; drives tumor invasion, angiogenesis, immunosuppression and chemoresistance
Dendritic cells (DC)	Type 1 conventional DC (cDC1)	Anti-tumor	CD141, CLEC9A, XCR1, IRF8	Flt3L/Flt3, IFN-γ/STAT1, glutamine metabolism pathway	Immunologically “hot tumors”, abundant T cell infiltration, presence of tertiary lymphoid structures	Favorable prognosis; core predictor of ICB response, the only subset that efficiently cross-presents antigens to activate CD8^+^ T cells
	Type 2 conventional DC (cDC2)	Predominantly anti-tumor	CD1c, CD11b, SIRPα, IRF4	GM-CSF/STAT5, Notch signaling	Inflammatory TME, CD4^+^ T cell-enriched regions	Favorable prognosis in most tumors; activates CD4^+^ helper T cells to amplify anti-tumor immune responses
	Plasmacytoid DC (pDC)	Functional heterogeneity	CD123, CD303, TLR7/9, IRF7	TLR7/9/IRF7, type I interferon signaling	Function dependent on TME status; functionally exhausted in immunosuppressive TME	Heterogeneous value; functional pDCs secreting IFN-α indicate favorable prognosis; high intratumoral infiltration correlates with poor prognosis in some tumors
Tumor-associated neutrophils (TAN)	N1-polarized neutrophils	Anti-tumor	CD54, CD86, HLA-DR, CD101	IFN-β, TNF-α/NF-κB	Acute inflammatory TME, early-stage tumors	Favorable prognosis; directly kills tumor cells via ROS, NETs and ADCC effects
	N2-polarized neutrophils	Pro-tumor	CD11b, VEGF-A, MMP-9, PD-L1, CXCR4, Arg-1	TGF-β/Smad, IL-6/STAT3	Chronic inflammatory TME, hypoxic TME, advanced metastatic stage	Unfavorable prognosis; promotes angiogenesis, matrix remodeling, immunosuppression and distant metastasis
Natural killer (NK) cells	Cytotoxic NK cells	Anti-tumor; functionally exhausted in TME	CD56, CD16, NKp46, NKG2D, KIR family	NKG2D/ligand, IL-15/STAT5; TGF-β/Smad	Immune surveillance phase, early-stage tumors; dysfunctional in hypoxic/immunosuppressive TME	High intratumoral infiltration indicates favorable prognosis; exhausted phenotype (low cytotoxicity, high inhibitory receptors) indicates poor prognosis
Innate lymphoid cells (ILC)	ILC1	Predominantly anti-tumor; pro-tumor in some contexts	Lin^-^CD45^+^CD127^+^T-bet^+^, CD49a, NKp44^-^	IL-12/STAT4; TGF-β	Epithelial tumors (e.g., gastrointestinal tumors, bladder cancer)	Functional ILC1 indicate favorable prognosis; TGF-β-induced aberrant phenotype indicates tumor progression risk
	ILC2	Predominantly pro-tumor	Lin^-^CD45^+^CD127^+^CRTH2^+^, GATA3^+^, c-Kit	IL-25/IL-33/STAT6, IL-13 pathway	Th2-inflammatory TME, breast cancer, lung cancer	Poor prognosis in most cases; recruits M2 macrophages via IL-13 and promotes tissue fibrosis
	ILC3	Predominantly pro-tumor; anti-tumor in mucosal tumors	Lin^-^CD45^+^CD127^+^RORγt^+^, NKp44, CD117	IL-23/STAT3, IL-22 pathway	Mucosa-associated tumors (e.g., gastrointestinal tumors, cervical cancer)	Poor prognosis in most cases; IL-17 suppresses CD8^+^ T cell function; exerts anti-tumor effects via mucosal barrier protection
γδ T cells	Vδ1/Vγ9Vδ2 subsets	Predominantly anti-tumor	TCRγδ, Vγ9Vδ2 (peripheral), Vδ1	BTN2A1/BTN3A1 (phosphoantigen recognition), IFN-γ pathway	Mucosal tumors, solid tumor stroma; phosphoantigen-enriched TME	High infiltration of Vδ1^+^ cells indicates favorable prognosis; exerts anti-tumor effects via direct cytotoxicity and immunomodulation
	γδT17 cells	Pro-tumor	RORγt^+^, IL-17^+^	IL-23/STAT3, RORγt transcriptional regulation	Chronic inflammatory TME, colorectal cancer, etc.	Unfavorable prognosis; secretes IL-17 to promote angiogenesis and recruit MDSCs and neutrophils

Although the classical M1/M2 dichotomous model holds significant conceptual and *in vitro* value, its limitations in accurately depicting the highly complex *in vivo* microenvironment have become increasingly apparent with the widespread application of high-resolution technologies such as single-cell RNA sequencing (scRNA-seq) and spatial transcriptomics (31). In the authentic solid tumor microenvironment, TAMs rarely exist in mutually exclusive, pure M1 or M2 states. Instead, they exhibit a highly dynamic and continuous phenotypic spectrum, frequently adopting “chimeric” states that simultaneously express both pro-inflammatory and anti-inflammatory markers (26). This plasticity makes it difficult for traditional polarization paradigms to precisely capture their complex biological behaviors. Consequently, the immunological field is gradually moving beyond traditional polarization labels toward a new generation of multi-dimensional classification systems that integrate transcriptomic signatures, cellular ontogeny, and spatial niches (32).

At the transcriptomic level, researchers increasingly define heterogeneous TAM subpopulations and their specific functions based on core molecular markers (32). For ^+^instance, SPP1^+^ TAMs, widely identified across various solid tumors, primarily mediate angiogenesis and extracellular matrix remodeling, whereas TREM2 and APOE^+^ TAMs are closely associated with profound immunosuppression and lipid metabolic reprogramming (33). Regarding cellular ontogeny, the emerging framework distinguishes TAMs into embryonic-derived tissue-resident macrophages (TRMs) and peripherally recruited monocyte-derived macrophages (Mo-Macs). Studies indicate that TRMs often participate in constructing profibrotic and immunosuppressive barriers during early tumorigenesis, while Mo-Macs demonstrate substantial transcriptomic plasticity as the tumor progresses. Furthermore, based on spatial niches, recent research highlights the decisive role of the physical microenvironment in dictating macrophage fate. TAMs located at the invasive margin, within the hypoxic core, or in perivascular regions exhibit fundamentally distinct metabolic states and immune regulatory networks. In summary, this novel classification paradigm, grounded in multi-dimensional features, not only accurately reconstructs the true *in vivo* heterogeneity of TAMs but also provides a more translatable theoretical framework for developing precision immunotherapies targeting specific pro-tumoral macrophage subsets (32).

In promoting metastasis, TAMs secrete matrix metalloproteinases (MMPs), especially MMP-9 and MMP-2, which degrade the extracellular matrix (ECM) and disrupt basement membrane integrity. This degradation compromises physical barrier to tumor cell invasion into surrounding tissues (34, 35). Furthermore, TAMs can secrete epidermal growth factor (EGF), establishing the EGF-CSF-1 paracrine loop with tumor cells that enhances, the motility and invasiveness of tumor cells. This interaction can even promote epithelial-mesenchymal transition (EMT), conferring stem-like properties and drug resistance upon tumor cells (35, 36).

At the molecular level, TAM polarization is tightly governed by intracellular signaling networks and metabolic reprogramming. While the NF-κB and STAT1 pathways typically drive M1 polarization, STAT3 and STAT6 cascades primarily mediate the M2 phenotype. For instance, tumor-derived IL-6 activates STAT3 signaling in macrophages, upregulating anti-inflammatory gene transcription (37). Metabolic reprogramming further dictates macrophage fate: M1 macrophages rely on aerobic glycolysis and an active pentose phosphate pathway to support cytotoxic molecule synthesis, whereas M2 macrophages preferentially utilize fatty acid oxidation (FAO) and mitochondrial oxidative phosphorylation (OXPHOS) to sustain pro-repair functions (38). Within the TME, lactate accumulation, altered lipid metabolism, and glucose competition serve as metabolic checkpoints that direct macrophage polarization toward the pro-tumorigenic M2 state (39).

Macrophages within the TME are integral components of tumor ecosystem. The dynamic balance between their M1 anti-tumor effects and M2 pro-tumor activities directly determines the nature of the tumor immune microenvironment (40). During early tumorigenesis, M1-like macrophages typically predominate, acting to clear malignant cells. However, in advanced tumors, the educating effects of the microenvironment drive TAMs to predominantly adopt an M2-like phenotype, establishing an immunosuppressive barrier that promotes tumor growth, angiogenesis, and metastasis. Consequently, elucidating the regulatory mechanisms governing TAM plasticity holds substantial translational potential for developing novel immunotherapies aimed at re-polarizing TAMs from a pro-tumorigenic M2 state back to a tumor-suppressive M1 phenotype (41).

### 2.2 Natural killer cells

NK cells are integral components of the innate immune system, serving as the first line of defense against viral infections and malignant tumors (42). Unlike adaptive T and B lymphocytes, NK cells can directly eliminate transformed cells through their unique receptor recognition mechanisms without requiring antigen pre-sensitization. Despite their potent cytotoxic activity, their infiltration capacity is frequently restricted, and their functions are subject to profound exhaustion within the complex TME (43, 44). Elucidating the mechanisms by which the TME shapes NK cell phenotypes and suppresses their functions is essential for understanding tumor immune evasion and developing novel immunotherapeutic strategies (45).

The activation of NK cells is governed by a dynamic balance between signals from activating and inhibitory receptors (46, 47). Under physiological conditions, healthy cells express high levels of major histocompatibility complex class I (MHC-I) molecules. These molecules primarily engage inhibitory receptors on NK cells, such as killer immunoglobulin-like receptors (KIRs) or the CD94/NKG2A heterodimer, transmitting a “self” signal that suppresses NK cell-mediated cytotoxicity (48). During early tumorigenesis, malignantly transformed cells often downregulate MHC-I expression to evade T cell attack. This “missing-self” state releases the inhibition on NK cells. Concurrently, tumor cells upregulate stress-induced ligands such as MICA/B and ULBPs, which bind to activating receptors on NK cells, and trigger an “induced non-self” signal. Upon activation, NK cells release perforin and granzymes or induce target cell apoptosis via the Fas/FasL pathway. However, as the tumor progresses, the TME evolves into a highly immunosuppressive niche that, employs multiple mechanisms to systemically undermine the anti-tumor efficacy of NK cells (49). The physicochemical characteristics of the TME represent primary factors constraining NK cell function (50). Solid tumors frequently exhibit severe hypoxia, acidosis, and nutrient deprivation. In particular, the overexpression of hypoxia-inducible factor 1-alpha (HIF-1α) in tumor cells not only promotes angiogenesis but also drives intrinsic metabolic reprogramming (51). Tumor cells avidly consume glucose and generate high concentrations of lactate, leading to marked depletion of glucose within the microenvironment. Because activated NK cells heavily rely on glycolysis to sustain their cytotoxic functions and cytokine production, glucose deprivation directly results in the metabolic suppression of NK cells (52, 53). Furthermore, elevated lactate concentrations cause acidification of the microenvironment, which not only inhibits NK cell proliferation but also downregulates surface activating receptors (e.g., NKp46 and NKG2D) and impairs the release of perforin and granzymes (54, 55). This metabolic competition constitutes a significant physicochemical barrier through which the TME suppresses NK cells. Beyond the pressures of the physicochemical environment, the accumulation of immunosuppressive cell populations within the TME plays a crucial role in NK cell inactivation. Tregs, MDSCs, and TAMs establish an intricate immunosuppressive network via cell-cell contact and the secretion of inhibitory cytokines (56). For instance, Tregs suppress NK cell activation by secreting TGF-β (57). As a key immunosuppressive mediator in the TME, TGF-β downregulates activating receptors (e.g., NKG2D and NKp30) on NK cells. It also suppresses the mTOR signaling pathway, thereby impairing cellular metabolism and IFN-γ secretion (58). Furthermore, prolonged exposure to TGF-β can drive the transdifferentiation of cytotoxic NK cells into ILC1-like cells with reduced cytotoxicity (59), or shift them toward a pro-angiogenic phenotype (60). Concurrently, MDSCs deplete L-arginine in the microenvironment via Arg-1 and generate reactive oxygen species (ROS), thereby suppressing NK cell cytotoxicity and downregulating activating receptors as well as their associated CD3ζ signaling subunit. This disruption consequently blocks the signal transduction from activating receptors (61, 62) (**Table 1**).

Tumor-intrinsic immunoediting and evasion mechanisms also play critical roles (15). To evade recognition by NK cells, tumor cells utilize proteolytic enzymes to shed surface NKG2D ligands, such as MICA/B, releasing soluble fragments (63). These soluble ligands serve as decoys, that competitively bind to NKG2D on NK cells. This interaction triggers receptor internalization and degradation, thereby desensitizing NK cells to tumor targets (64). Furthermore, tumor cells frequently upregulate the expression of HLA-E molecules. HLA-E exhibits a strong binding affinity for the inhibitory receptor NKG2A/CD94 on NK cells, which delivers a potent inhibitory signal that directly paralyzes the cytotoxic machinery of NK cells (65). Through such receptor-ligand interactions, tumor cells can effectively evade NK cell-mediated surveillance and induce a state of immune tolerance (63). Within the TME, NK cells confront an immunosuppressive milieu driven by hypoxia, metabolic constraints, inhibitory cells, and soluble factors. This environment not only restricts the physical infiltration of NK cells but also significantly alters their phenotype and function, leading to immune escape. However, with a growing understanding of TME-mediated immunosuppression, therapeutic strategies focused on reversing NK cell exhaustion and enhancing their metabolic fitness are rapidly advancing (53). Future research should further focus on precisely modulating the metabolic-immune axis and employing combination therapies to overcome the physical barriers of solid tumors (66). Such approaches are essential to fully unleash the immense potential of NK cells in cancer immunotherapy, thereby improving long-term clinical outcomes for cancer patients.

### 2.3 Neutrophils

Neutrophils are the most abundant leukocytes in peripheral blood and have historically been regarded as a non-specific first line of defense against bacterial and fungal infections. Consequently, their role in tumor immunity was long underappreciated (67). However, with advanced characterization of the TME, it is now evident that neutrophils serve as integral modulators in the processes of tumor initiation, progression, and metastasis (68, 69).

Driven by specific signals within the TME, neutrophils infiltrating tumor tissue-termed TANs-exhibit remarkable phenotypic plasticity and functional heterogeneity (70). This heterogeneity is reflected not only in the complexity of their recruitment mechanisms but, more significantly, in their dual role: they can exert anti-tumor effects, yet under specific conditions, transform into “accomplices” that promote tumor progression. This dual nature profoundly impacts the prognosis of cancer patients and the efficacy of immunotherapy (71). The recruitment of neutrophils to tumor sites is a tightly regulated process governed by a network of chemokines. Tumor cells and stromal cells within the microenvironment secrete chemokines such as CXCL1, CXCL2, CXCL5, and CXCL8, which bind to the CXCR2 receptors on the neutrophil surface (72). This interaction guides their release from the bone marrow and their directional migration to the tumor site.

Upon recruitment of the TME, neutrophils undergo polarization driven by the local cytokine milieu. Analogous to macrophage polarization, TANs were classically categorized into an anti-tumorigenic N1 phenotype and a pro-tumorigenic N2 phenotype. This dichotomy is dictated by microenvironmental signals, with IFN-β and TGF-β driving N1 and N2 polarization, respectively. While N1 neutrophils may predominate during early tumor development and exert direct cytotoxicity, advancing tumors establish a TGF-β-rich immunosuppressive milieu that skews TANs toward the N2 phenotype N2 TANs facilitate malignant progression primarily via angiogenesis and immunosuppression. They secrete matrix metalloproteinase-9 (MMP-9) to degrade the extracellular matrix, releasing matrix-bound VEGF to stimulate neovascularization. Concurrently, immunosuppressive TANs restrict T-cell function by depleting microenvironmental arginine Arg-1. Furthermore, TAN-derived ROS and nitric oxide (NO) induce T-cell receptor (TCR) nitration to impair antigen recognition, while high PD-L1 expression directly blunts cytotoxic T lymphocyte activity, forming a robust barrier against adaptive immunity (**Table 1**).

However, recent advances in single-cell transcriptomics indicate that the traditional N1/N2 binary model oversimplifies the profound heterogeneity of TANs (73). The contemporary consensus conceptualizes neutrophil polarization as a continuous spectrum rather than discrete states (74). Modern nomenclature frameworks have shifted toward defining TAN subpopulations based on high-dimensional transcriptomic profiles, developmental ontogeny (75) (e.g., mature vs. immature/MDSC-like trajectories), spatial tissue localization, and specific functional modules (such as antigen-presenting or metabolically exhausted phenotypes). This multi-dimensional profiling provides a higher-resolution paradigm to accurately decipher TAN biology and identify novel targets for precision innate immunotherapy (76).

The role of Neutrophil Extracellular Traps (NETs) in tumor metastasis has emerged as a critical area of investigation. While physiologically acting to sequester pathogens, this innate defense network is pathologically co-opted by malignant cells within the tumor milieu (77). Research indicates that circulating tumor cells (CTCs) can be entrapped by intravascular NETs. This not only protects CTCs from shear stress in the bloodstream and killing by NK cells but also facilitates their extravasation through vessel walls at distant organs, leading to the formation of metastatic niches (78). Furthermore, NET-associated proteases can cleave laminin in the extracellular matrix, unveiling cryptic epitopes that can activate dormant cancer cells and initiate proliferation programs. Consequently, NETs function beyond mere physical tethers during dissemination. They actively establish a growth-permissive niche that drives the exit of tumor cells from dormancy and facilitates their subsequent colonization (79). Despite the pro-tumorigenic roles demonstrated by N2-type neutrophils and NETs, the therapeutic potential of N1-type neutrophils remains a vital avenue for anti-tumor strategies. N1-type neutrophils exert direct cytotoxicity against tumor targets through the generation of ROS, NO, and proinflammatory cytokines, including TNF-α (74). Furthermore, they serve as key effector cells in antibody-dependent cellular cytotoxicity (ADCC), thereby augmenting tumor clearance during monoclonal antibody therapies (80, 81). Therefore, therapeutic paradigms have shifted from systemic neutrophil depletion toward targeted cellular reprogramming (82). Pharmacological agents targeting TGF-β signaling or the CXCR2 chemotactic axis are currently undergoing clinical evaluation (76). These approaches aim to block the recruitment of pro-tumorigenic TANs or repolarize them toward an anti-tumorigenic N1 phenotype, thereby restoring neutrophil-mediated anti-tumor immunity (83, 84).

Neutrophils within the tumor microenvironment represent a highly dynamic and functionally dichotomous cell population. While promoting tumor angiogenesis and immune escape, they retain the capacity to exert potent anti-tumor effector functions. Elucidating the molecular switches governing TAN polarization and the specific mechanisms underlying NET formation is crucial for understanding the systemic impact of tumors. Future research, if successful in achieving precise phenotypic manipulation of neutrophils, will undoubtedly provide new breakthroughs in overcoming the immune tolerance of solid tumors and preventing distant metastasis.

### 2.4 Dendritic cells

DCs represent the most potent professional antigen-presenting cells (APCs), serving as an essential functional bridge between innate and adaptive immunity (85, 86). During physiological tumor immune surveillance, DCs capture and process TAAs, load them onto MHC molecules, and migrate to tumor-draining lymph nodes to prime naïve T lymphocytes, thereby initiating antigen-specific anti-tumor immune responses (87). However, the quantity, phenotype, and function of DCs often undergo significant alterations within the TME (85). Tumor-derived inhibitory factors and metabolic alterations systematically impair DC maturation, dampening their immunostimulatory capacity and converting them into tolerogenic cells that induce immune tolerance-a critical mechanism of tumor immune escape (88). Tumor-infiltrating DCs are broadly categorized into conventional dendritic cells (cDCs) and plasmacytoid dendritic cells (pDCs). With cDCs further stratified into cDC1 and cDC2 lineages based on transcription factor dependency and surface marker profiles (87). Developmentally dependent on Batf3 and IRF8, cDC1s (phenotypically CD8α^+^/CD103^+^ in mice and CD141^+^ in humans) specialize in antigen cross-presentation, shuttling exogenous TAAs onto MHC class I molecules to activate CD8^+^ cytotoxic T lymphocytes (89). High intratumoral cDC1 abundance positively correlates with patient prognosis across solid cancers and serves as a major determinant of efficacy for immune checkpoint inhibitors (90). In contrast, cDC2 (typically CD11b^+^) primarily rely on IRF4 and are proficient at activating CD4^+^ helper T cells via the MHC class II pathway (91). pDCs are renowned for their robust production of type I interferons. However, within the tumor milieu, they are frequently skewed toward a tolerogenic phenotype that promotes the generation of Tregs (92).

The capacity of DCs to trigger anti-tumor immune responses, DCs frequently display compromised functional activity in the TME, a condition commonly referred to as “DC paralysis” (93). Multiple suppressive mechanisms existing in the TME contribute to this dysfunction. Firstly, tumor-derived soluble factors act as the first barrier to DC differentiation and maturation. VEGF, a major regulator of angiogenesis, also suppress the differentiation of hematopoietic progenitor cells into functional DCs, partially through the inhibition of the NF-κB pathway, potentially contributing to the accumulation of immature DCs (iDCs) *in vivo (*94). These iDCs typically display low levels of MHC and co-stimulatory molecules such as (CD80 and CD86), thereby limiting their capacity to deliver the signals required for efficient T cell activation (95). Instead, they frequently upregulate inhibitory molecules (such as PD-L1), which induce T cell anergy or apoptosis. Furthermore, inhibitory cytokines such as IL-10 and TGF-β further block maturation signals for DCs, which locks them into a persistent tolerogenic state (Tolerogenic DCs) (96).In recent years, metabolic reprogramming has increasingly been recognized as one of a key mechanisms underlying DC dysfunction within the TME (97). Among these pathways, dysregulated lipid metabolism has garnered particular attention. As the tumor microenvironment is often rich in necrotic cell debris and accumulated lipids, DCs take up substantial amounts of exogenous lipids via scavenger receptors, contributing to intracellular lipid droplet accumulation (98). This state of “lipid overload” not only compromises the assembly of the antigen processing machinery, but oxidized lipids activate the endoplasmic reticulum (ER) stress response. Subsequently, sustained activation through the IRE1α-XBP1 axis impairs the cross-presentation capability of DCs (99). Furthermore, the lactate-rich environment generated by the high glucose consumption of tumor cells lowers the intracellular pH within DCs (100). This acidic milieu inhibits their glycolytic metabolism, which impairs both their migratory capacity and cytokine secretion. This form of metabolic “competitive inhibition” fundamentally severs the energy supply required for DCs to perform their effector functions (21).Tumor cells can impede DC infiltration through specific signaling pathways, potentially contributing to the “immune desert” phenotype. For instance, certain melanoma cells suppress the expression of the chemokine CCL4 by activating the Wnt/β-catenin signaling pathway. As CCL4 serves a key factor for recruiting cDC1s to the tumor site, its suppression often results in a scarcity of cDC1 within the tumor core (101). Consequently, the recruitment of CD8^+^ T cells into the central tumor region is markedly restricted. This mechanism highlights how tumor cells can precisely disrupt the chemokine network to block the initiation of the cancer-immunity cycle at an early stage. In addition, physical factors within the TME, such as increased stromal stiffness, can impair DC function. Transduced into intracellular signals, these biomechanical cues affect pseudopodia formation and motility, thereby restricting DCs predominantly to the peripheral stroma and impeding their penetration into the tumor core (102).

DCs occupy a pivotal hub within the tumor microenvironment. While serving as key initiators of anti-tumor immunity, they are also highly susceptible to the immunosuppressive networks of the TME. Tumors employ various mechanisms to compromise DC function, ranging from impaired recruitment and restricted differentiation to metabolic dysregulation and signaling interference during maturation (103).

### 2.5 Innate lymphoid cells

ILCs are a subset of innate lymphocytes that lack antigen-specific receptors while possessing robust cytokine-secreting capabilities, positioning them as an active area of interest in TME research (104, 105). ILCs development largely depend on the transcriptional repressor Id2 and functionally parallel the T helper subsets (Th1, Th2, and Th17) of the adaptive immune system, constituting an innate “helper” branch (106). Unlike the primarily circulating NK cells, helper ILCs are primarily tissue-resident cells that are widely distributed across mucosal barriers and parenchymal organs (107). This strategic spatial distribution enables ILCs to rapidly sense metabolic shifts, cellular stress, and tissue damage signals (such as alarmins), thereby initiating immune responses or contributing to tissue remodeling during early stages of tumorigenesis (108). However, within the complex ecosystem of solid tumors, ILC activity is rarely uniform or strictly anti- or pro-tumorigenic. Instead, they display substantial functional heterogeneity and functional plasticity that depends on their subset classification and the specific cytokine network of the local microenvironment (17). Group 1 Innate Lymphoid Cells (ILC1s) are largely regulated by the transcription factor T-bet and are defined by their secretion of IFN-γ and TNF (109). Although ILC1s and NK cells are both classified within Group 1, they display distinct transcriptional dependencies and cytotoxic profiles. While NK cells characteristically rely on Exomes and exhibit robust perforin/granzyme-mediated cytotoxicity, ILC1s tend to act predominantly in a helper-like capacity (110). In certain tumor models, IFN-γ produced by ILC1s can induce the upregulation of MHC class I molecules on tumor cells and promote M1 macrophage polarization, potentially contributing to an anti-tumor inflammatory microenvironment (111). In TGF-β-rich tumor microenvironments, cytotoxic NK cells can undergo phenotypic conversion by downregulating Exomes and upregulating tissue-resident markers such as CD49a and CD103 transdifferentiate into low-cytotoxicity ILC1-like cells (59). This phenotypic transition not only diminishes the pool of effective cytotoxic cells within the tumor, but can also trigger TNF secretion, which may promote tumor vascular permeability and metastasis under specific conditions. Consequently, an expanded ILC1 population within the TME is often regarded as a marker of NK cell functional deterioration, and it is associated with poor prognosis in various solid tumors (112).

Group 2 Innate Lymphoid Cells (ILC2s) largely rely on the transcription factor GATA3. They primarily respond to epithelial-derived cytokines such as IL-33, IL-25, and TSLP, producing IL-4, IL-5, IL-13, and Amphiregulin (108). ILC2s are key initiators of type 2 immune responses in allergic diseases and parasitic infections. However, their role in the context of cancer is fraught with controversy and contradiction. On one hand, ILC2s can promote TAM polarization toward an immunosuppressive M2-like phenotype via IL-13 secretion and facilitate Treg expansion, which may dampen the anti-tumor activity of CD8^+^ T cells (113). Furthermore, ILC2-derived Amphiregulin can engage the EGFR signaling pathway on tumor cells, potentially promoting their proliferation and survival. Within the tumor context, this tissue-repair response can be co-opted to foster tumor growth and metastatic dissemination. Particularly in breast and gastric cancers, elevated ILC2 infiltration has been associated with accelerated disease progression (114). Conversely, ILC2s have also been observed to exhibit anti-tumor activity in specific models, such as lung cancer and melanoma. This is largely attributed to their secretion of IL-5, which recruits and activates eosinophils capable of mediating tumor cell clearance through cytotoxic granule release (115). Therefore, ILC2s function is highly dependent on the specific organ microenvironment and prevailing cytokine milieu. Group 3 Innate Lymphoid Cells (ILC3s) depend on the transcription factor RORγt and primarily produce IL-17 and IL-22, playing a key role in maintaining intestinal mucosal homeostasis. Within the tumor microenvironment, ILC3s also exhibit a nuanced, dual role with both pro- and anti-tumorigenic potential (108). As members of the lymphoid tissue inducer (LTi) cell family, ILC3s express lymphotoxin and adhesion molecules, playing a key role in facilitating the formation of Tertiary Lymphoid Structures (TLS). TLSs are ectopic lymphoid aggregates typically residing within the tumor stroma or periphery and serve as important sites for the priming and expansion of anti-tumor T cells. Consequently, tumors rich in LTi-like ILC3s are often associated with better T cell infiltration and improved patient survival (116). However, the effector molecules secreted by ILC3s can also exert pro-tumorigenic effects depending on the context. For instance, IL-22 has been shown to promote the proliferation and inhibit the apoptosis of colorectal cancer cells by activating the STAT3 signaling pathway (117). Meanwhile, IL-17 exhibits pro-angiogenic properties and can recruit neutrophils, which fosters a chronic inflammatory environment that drives tumor progression (118). Furthermore, studies have found that certain tumor cells can stimulate ILC3s to produce these pro-tumorigenic factors by secreting IL-23, establishing a detrimental positive feedback loop (119).

A defining feature underlying the functions of various ILCs is their high degree of plasticity within the TME. Rather than remaining static, the tumor microenvironment represents a dynamic ecosystem characterized by metabolic stress and complex cytokine networks (120). Beyond the conversion of NK cells to ILC1s, ILC3s can downregulate RORγt while acquiring T-bet in environments that lack IL-23 or are rich in IL-12/IL-18. This change allows them to transdifferentiate into “ex-ILC3s” or ILC1-like cells, thereby acquiring the capacity to secrete IFN-γ (121). This plasticity suggests that the ILC phenotypes observed in a single tumor biopsy may represent a transient snapshot; their functional states may shift significantly as the tumor evolves. Such inherent plasticity presents both a challenge and an opportunity for ILC-targeted immunotherapies: if pharmacological interventions can inhibit signals like TGF-β that drive pro-tumorigenic phenotypic shifts, or harness cytokines like IL-12 to induce their reversion towards an anti-tumor phenotype, it may be possible to restore the surveillance function of the innate immune system (17).

Innate lymphoid cells construct a complex and dynamic, regulatory network within the tumor microenvironment. Yet they are also vulnerable to being co-opted by malignant cells. Currently, specific clinical therapies targeting ILCs are still in the exploratory stage, with a major challenge being the lack of surface markers that can reliably distinguish ILCs from their corresponding T cell subsets. This means broad cytokine blockade strategies may carry a risk of unintended systemic side effects. Future research should leverage to employ high-throughput technologies, such as single-cell sequencing, to further delineate the heterogeneity map of ILCs in human tumors and elucidate the critical molecular switches that govern their phenotypic transitions. This will pave the way for establishing a new generation of tumor immunotherapy strategies that are centered on reprogramming ILCs. These strategies aim to restore these tissue-resident populations as effective mediators.

### 2.6 γδ T cells

Historically, tumor immunology research has predominantly focused on conventional αβ T cells,. However, with an expanding appreciation of the complexity of the TME, the functions of γδ T cells have become a focal point of investigation (122). γδ T cells constitute only 1%-5% of total peripheral blood lymphocytes but are highly enriched in epithelial tissues and mucosal barriers. As a bridge between innate and adaptive immunity, γδ T cells possess a series of unique biological characteristics, most notably their ability to recognize antigens independently of MHC presentation (123). This feature enables them to maintain effective immune surveillance capability even when tumor cells downregulate MHC molecules-a common evasion mechanism to escape CD8^+^ T cell killing (18). Within the TME, γδ T cells demonstrate considerable functional plasticity, serving as potent cytotoxic effectors to inhibit tumor progression. However, under alternative microenvironmental influences, they can be induced to foster inflammation and immune suppression (124).

Based on the variable region gene usage of the TCR δ chain, human γδ T cells are broadly classified into two major subsets: Vδ2 T cells and Vδ1 T cells. Vδ2 T cells are predominantly found in peripheral blood and are typically paired with the Vγ9 chain (123). They recognize non-peptide phosphoantigens, such as isopentenyl pyrophosphate (IPP), which are produced by metabolic dysregulation in tumor cells. Malignant transformation often leads to aberrantly elevated intracellular IPP levels due to dysregulation of the mevalonate pathway. This metabolic signal is specifically recognized by Vδ2 T cells through the butyrophilin 3A1 (BTN3A1) and BTN2A1 complex on the cell surface (125). In contrast, Vδ1 T cells are mainly tissue-resident, located in the skin, intestine, and within solid tumors (126). Their recognition mechanisms are broader and less fully understood, they mainly recognize stress-induced ligands (such as MICA/B and ULBP) on tumor cells via receptors like NKG2D, or lipid antigens presented by CD1c/d (127). These differences in subset distribution and recognition mechanisms broadly underlie their functional heterogeneity within the TME.

Regarding anti-tumor immunity, activated γδ T cells exhibit cytotoxic activity comparable to or exceeding that of NK cells and CTLs. They can directly lyse tumor cells by releasing perforin and granzyme B, or induce target cell apoptosis through high expression of FasL and TRAIL (128). More uniquely, γδ T cells-especially the Vδ2 subset-express high levels of CD16, enabling them to mediate ADCC. This means they can synergize with therapeutic monoclonal antibodies to enhance clinical efficacy (129). Beyond direct killing, γδ T cells also function in a manner like APCs. It has been shown that activated γδ T cells can internalize tumor antigens, upregulate MHC class II molecules and costimulatory molecules (CD80/CD86), and cross-present antigens to CD4^+^ and CD8^+^ T cells, thereby facilitating downstream adaptive immune responses (130). It is notable that prototypical antitumor γδ T cells predominantly secrete IFN-γ and TNF-α, which are Th1-type cytokines critical for maintaining an inflammatory state in the TME, inhibiting angiogenesis, and upregulating the immunogenicity of tumor cells (18).

However, the role of γδ T cells in the TME is not exclusively anti-tumorigenic. A growing body of clinical data and experimental evidence indicates that γδ T cells can also exhibit immunosuppressive functions (131). Exposed to specific cytokine milieus, such as those enriched in IL-1β, IL-6, IL-23, and TGF-β, γδ T cells may undergo polarization and polarize toward a γδ T17 phenotype, which primarily secretes IL-17 (18). This pro-tumor phenotype is particularly common in colorectal cancer, breast cancer, and pancreatic cancer. IL-17 not only directly promotes the proliferation and survival of tumor cells but also induces surrounding stromal cells to secrete VEGF and MMP-9, thereby facilitating tumor angiogenesis (132). Furthermore, γδ T17 cells are key mediators of chronic inflammation in the TME. By secreting chemokines such as CXCL8, they recruit large numbers of MDSCs into tumor tissues (133). These MDSCs release ROS and Arg-1, impairing the antitumor function of Vδ2 T cells. Meanwhile, tissue-resident Vδ1 T cells often exhibit regulatory or pro-tumor properties in various solid tumors, and their infiltration density frequently correlates with poor clinical outcomes (134).

Owing to their unique antigen recognition mechanisms and potent cytotoxic potential, immunotherapies based on γδ T cells have become a major focus of current drug development. Current therapeutic strategies mainly *in vivo* activation and adoptive transfer of ex vivo expanded cells. Drugs such as bisphosphonates (e.g., zoledronic acid) can be employed to inhibit the mevalonate pathway, leading to intracellular accumulation of IPP in tumor cells and thereby specifically activating Vδ2 T cells *in vivo*. This approach has shown some efficacy in clinical trials (135). On the other hand, because γδ T cells recognize antigens independently of MHC presentation, they carry a minimal risk of graft-versus-host disease (GVHD). This characteristic makes them attractive candidates for developing allogeneic off-the-shelf cellular therapies (136). Currently, chimeric antigen receptor-engineered γδ T cells (CAR-γδ T) are being extensively tested in both hematological malignancies and solid tumors, aiming to combine the precise targeting of CARs with the multifaceted killing mechanisms of γδ T cells (137).

γδ T cells within the tumor microenvironment constitute a highly dynamic and functionally complex population. They serve as pivotal mediators of innate-like immune surveillance, offering broad-spectrum and rapid antitumor capabilities. However, they also remain susceptible to reprogramming by inflammatory networks in the TME. Under such conditions, they can adopt pro-angiogenic and immunosuppressive roles that facilitate tumor progression. A deeper understanding of the mechanisms driving the functional differentiation of distinct γδ T cell subsets in specific tumor contexts is crucial for optimizing current immunotherapy strategies. This is particularly true for the molecular switches that control IL-17 production and the pro-tumor phenotype (138). If future translational research can successfully mitigate their tumor-promoting risks while fully unleashing their MHC-independent cytotoxic potential, γδ T cells are poised to occupy cornerstone of next generation in the next decade of tumor immunotherapy.

## 3 Mechanistic pathways and crosstalk in TME

### 3.1 Key signaling pathways

In the complex TME landscape, the phenotypic plasticity and functional heterogeneity of innate immune cells are orchestrated by several pivotal intracellular signaling pathways (104). Among these, the cGAS-STING pathway shapes type I interferon responses and overall immunogenicity (139), the NF-κB/STAT3 network mediates the interplay between pro-inflammatory and immunosuppressive signaling (140), and the PD-1/PD-L1 axis promotes functional impairment and innate immune dysfunction (141). Together, these pathways represent crucial molecular nodes dictating the balance between innate immune surveillance and tumor immune evasion.

cGAS-STING Pathway: Regulating Type I Interferon and Immunogenicity The cGAS-STING pathway is a fundamental innate immune sensing mechanism responsible for detecting aberrant cytosolic DNA (142). In the context of the TME, this pathway plays a dual role in modulating tumor immunogenicity. During the early stages of anti-tumor immunity, cGAS recognizes tumor-derived double-stranded DNA (dsDNA) and catalyzes the synthesis of cyclic GMP-AMP (cGAMP), which subsequently activates endoplasmic reticulum-resident STING. This activation triggers the TBK1/IRF3 signaling cascade, culminating in the robust production of type I IFNs and various pro-inflammatory cytokines (143). Such a milieu is considered crucial for the activation and maturation of DCs, enhancing their antigen cross-presentation capabilities and the subsequent priming of CD8^+^ cytotoxic T lymphocytes (CTLs) (144). However, the cGAS-STING pathway also exhibits a paradoxical pro-tumoral effect (145). Chronic and persistent STING activation within the TME has been associated with the expression of immunosuppressive factors, such as IDO and IL-10, which may foster the recruitment of Tregs and the polarization of TAMs toward a pro-tumoral M2 phenotype (143). Furthermore, advanced malignancies frequently employ epigenetic silence mechanisms to downregulate cGAS or STING expression, effectively dismantling this innate sensing network to achieve immune escape (146) (**Figure 2**).

**Figure 2:**
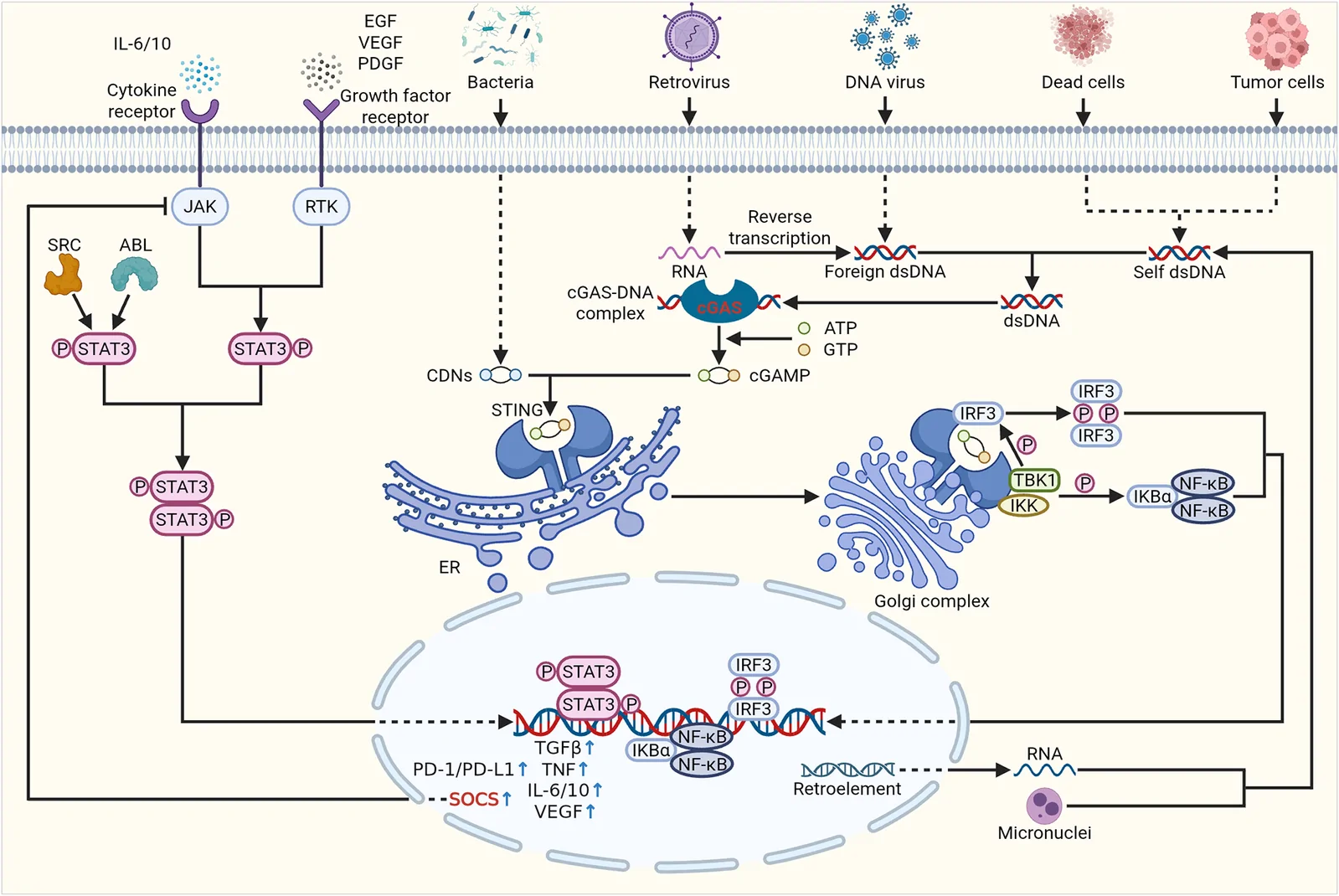
Schematic representation of the cGAS-STING, JAK-STAT3 and NF-κB signaling pathways in response to extracellular and intracellular stimuli. This diagram illustrates the integrated signaling networks triggered by various stimuli, including cytokines, growth factors, and exogenous or endogenous DNA. The JAK-STAT3 pathway is activated via cytokine receptors (e.g., IL-6/10) or growth factor receptors (RTK), as well as non-receptor tyrosine kinases like SRC and ABL, leading to the phosphorylation, dimerization, and nuclear translocation of STAT3. Simultaneously, the cGAS-STING pathway senses double-stranded DNA (dsDNA) from viruses (DNA viruses/retroviruses), bacteria, or self-DNA (from dead cells, tumor cells, or micronuclei). Upon dsDNA binding, cGAS catalyzes the production of cGAMP, which activates STING at the endoplasmic reticulum (ER). STING then translocates to the Golgi complex to recruit TBK1 and IKK, resulting in the activation and nuclear entry of IRF3 and NF-κB. Within the nucleus, these transcription factors synergistically regulate the expression of genes involved in immune evasion (e.g., PD-1/PD-L1), inflammatory responses (e.g., TNF, IL-6/10), and feedback inhibition (e.g., SOCS).

NF-κB/STAT3 Network: Coordinating Inflammation and Immunosuppression The crosstalk between Nuclear Factor kappa B (NF-κB) and Signal Transducer and Activator of Transcription 3 (STAT3) forms a core transcriptional network that coordinates the delicate balance between inflammation and immune tolerance in the TME (147). Under physiological conditions, canonical NF-κB signaling is a primary driver of the anti-tumoral M1 macrophage phenotype, characterized by the secretion of pro-inflammatory cytokines such as TNF-α and IL-12 (148). However, the persistent chronic inflammatory state of the TME can substantially alter this paradigm. Tumor-derived soluble factors, notably IL-6, potently activate the STAT3 signaling pathway in myeloid cells (149). STAT3 hyperactivation not only drives the repolarization of macrophages toward the immunosuppressive M2 phenotype but also transcriptionally upregulates various anti-inflammatory and pro-angiogenic genes. Consequently, these “educated” TAMs secrete abundant TGF-β and IL-10, establishing a robust biochemical barrier that impairs effector T cell functions (150). Additionally, the aberrant crosstalk between NF-κB and STAT3 is increasingly recognized as a key driver for the expansion and suppressive maintenance of MDSCs (151). This transcriptional network effectively rewires the metabolic and functional landscape of the stroma, reinforcing a pro-tumoral inflammatory state that facilitates tumor survival and metastasis (152) (**Figure 2**).

PD-1/PD-L1 Axis: Mediating Immune Exhaustion and Innate Dysfunction The PD-1 and PD-L1 constitute a well-established immune checkpoint axis historically recognized for mediating T cell exhaustion. However, its profound impact on innate immune dysfunction is increasingly appreciated (141). Within the complex TME, innate immune populations-particularly M2-like TAMs and immature DCs-frequently exhibit upregulated expression of PD-L1 (104). The engagement of PD-L1 with PD-1 receptors on infiltrating effector T cells delivers potent inhibitory signals, accelerating T cell exhaustion, anergy, and apoptosis (153). Beyond direct cell-to-cell contact, tumor cells and suppressive stroma actively release PD-L1-enriched exosomes, which act as systemic molecular messengers to attenuate immune activation both locally and systemically (154, 155). Emerging evidence indicates that PD-1 is also significantly expressed on the surface of various innate immune cells, including macrophages and NK cells. The activation of the PD-1/PD-L1 axis directly dampens the phagocytic capacity of TAMs and impairs the cytotoxic degranulation of NK cells (156, 157). Therefore, the PD-1/PD-L1 signaling axis not only paralyzes adaptive immune responses but also fundamentally compromises the first line of innate immunological defense, making it a critical node for therapeutic intervention (**Figure 3**).

**Figure 3:**
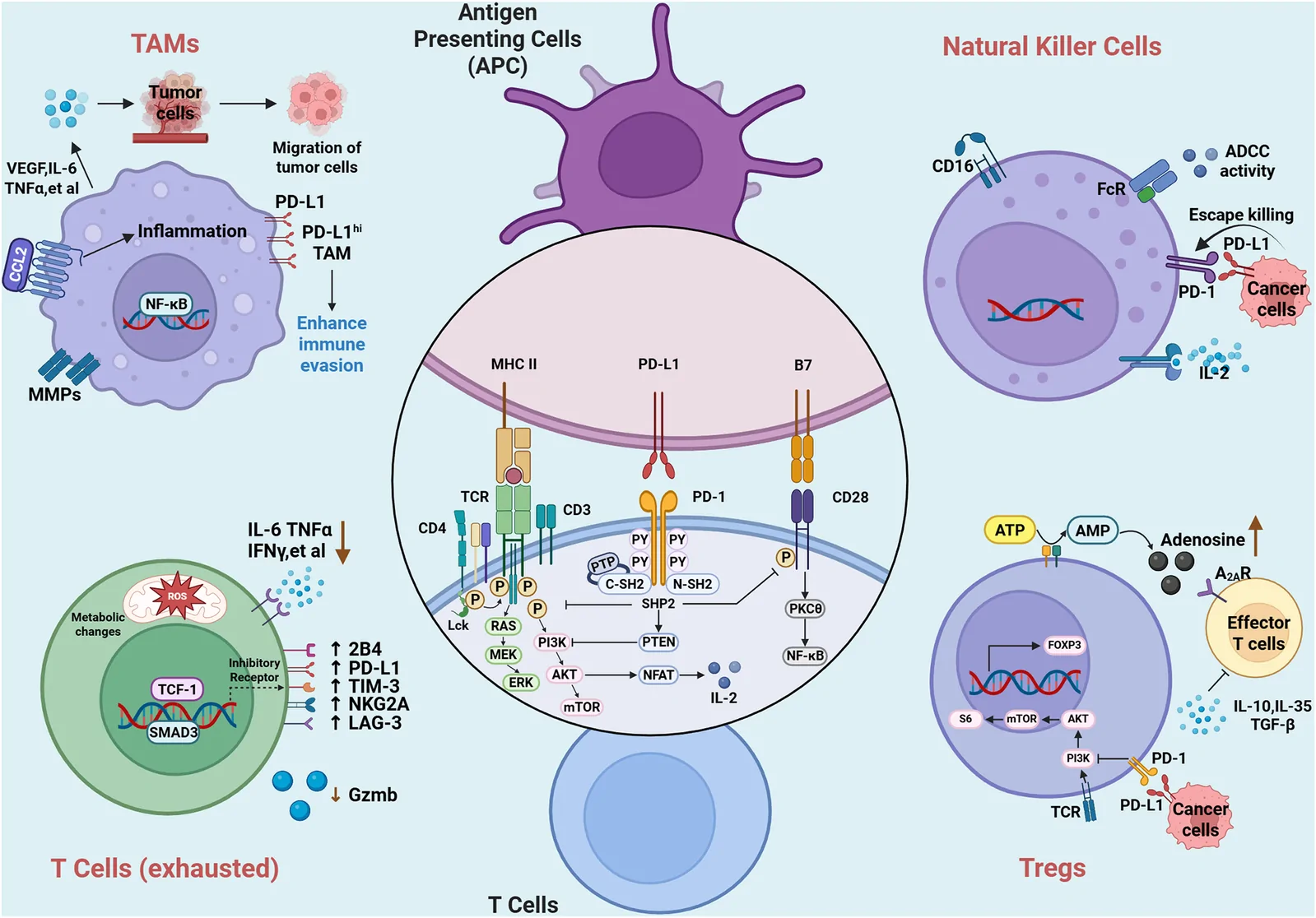
The immune evasion network in the tumor microenvironment and signaling mechanisms of the PD-1/PD-L1 Axis. (Left) The immune synapse model between APCs and T cells. Full activation of T cells relies on dual signals from the TCR and CD28, which drive the PI3K/AKT and RAS/MEK/ERK signaling cascades. The engagement of PD-1 with PD-L1 recruits the SHP2 phosphatase through the phosphorylation of its intracellular motifs, thereby blocking the aforementioned kinase pathways and subsequently inhibiting T cell activation and IL-2 production. (Right) Remodeling of key immune cell populations in the TME. Exhausted T cells exhibit metabolic stress (increased ROS), upregulated expression of inhibitory receptors (e.g., TIM-3, LAG-3), and impaired secretion of effector molecules (IFNγ, Gzmb). Tregs accumulate and suppress effector T cells by producing adenosine and inhibitory cytokines (IL-10, TGF-β). TAMs secrete pro-inflammatory and pro-angiogenic factors (VEGF, IL-6) to promote tumor migration, and express high levels of PD-L1 to enhance overall immune evasion. Furthermore, PD-L1-expressing cancer cells can directly bind to PD-1 on NK cells, inhibiting their ADCC activity and allowing tumor cells to escape killing.

### 3.2 Metabolic reprogramming

During the initiation and progression of malignant tumors, metabolic reprogramming has been established as a core biological hallmark, closely linked to malignant phenotypes such as sustained proliferation, resistance to apoptosis, invasion, and metastasis (158). Within the highly heterogeneous niche of the TME, the supply of nutrients and oxygen is often limited, leading to metabolic competition and cooperation among different cellular subpopulations (159). This metabolic remodeling not only meets the biosynthetic and energy demands of rapidly proliferating tumor cells but also substantially alters the metabolic landscape of the microenvironment. Through mechanisms such as nutrient deprivation and the accumulation of specific metabolites, it reshapes the functional phenotypes of immune cells, thereby facilitating local immune tolerance and evasion (159, 160).

The dysregulation of glucose metabolism, known as the Warburg Effect, represents a key metabolic hallmark within the TME. Even under normoxic conditions, cancer cells preferentially utilize the less efficient glycolytic pathway to convert glucose into lactate, rather than engaging mitochondrial oxidative phosphorylation. This seemingly paradoxical deviation from energy-efficient principles is widely considered an adaptive mechanism that supports, tumor cells to adapt to the demands of rapid proliferation (161). On one hand, enhanced glycolytic flux supplies carbon skeletons required for the synthesis of nucleotides, amino acids, and lipids. On the other hand, it supports the generation of NADPH to maintain intracellular redox homeostasis. At the molecular level, this process is finely regulated by signaling axes involving HIF-1α, c-Myc, and PI3K/Akt/mTOR. These pathways lead to the upregulation of glucose transporters (e.g., GLUT1/3) and rate-limiting glycolytic enzymes such as hexokinase 2 (HK2) and pyruvate kinase M2 (PKM2) (162, 163).

However, the avid uptake of glucose by tumor cells leads to a state of “glucose deprivation” within the TME, which can compromise infiltrating Teff. Teff cell activation and cytokine production are highly dependent on glycolysis for energy. The low-glucose environment created by tumor cells forces T cells into metabolic maladaptation, thereby inhibiting mTORC1 activity and reducing the synthesis of effector molecules such as IFN-γ. Ultimately, this drives T cells into a state of anergy or exhaustion (21). Furthermore, the large amount of lactate produced by tumor glycolysis is exported out of the cells via monocarboxylate transporter 4 (MCT4), resulting in significant acidification of the TME. This acidic microenvironment not only directly suppresses the cytotoxic activity of T cells and NK cells but also acts as a signaling molecule (54). It promotes the polarization of TAMs toward the immunosuppressive M2 phenotype by inducing the expression of VEGF and Arg-1 (164). This “metabolic checkpoint” mechanism indicates that the Warburg effect is not merely an energy acquisition strategy for tumor cells but also an active process that reshape the immune microenvironment.

Building upon this metabolic checkpoint, the consequent extracellular acidification exerts a multifaceted influence on the cellular composition and function within the TME (165). Beyond the direct suppression of effector lymphocytes and the induction of M2 macrophage polarization, a low-pH microenvironment impairs the antigen-presenting capacity of DCs (166). Acidosis restricts DC maturation and downregulates the expression of co-stimulatory molecules, thereby attenuating the priming phase of the adaptive immune response (104). Concurrently, this acidic niche selectively confers a survival and functional advantage to immunosuppressive cellular subsets, notably Tregs and MDSCs (167). For instance, Tregs can utilize extracellular lactate as an alternative carbon source, which maintains their lineage stability and enhances their suppressive capacity under acidic conditions (166, 167). Furthermore, elevated extracellular proton concentrations disrupt intracellular pH homeostasis in effector T cells, leading to impaired TCR activation and downstream signaling cascades (165). Consequently, tumor-induced acidosis functions as a critical biochemical barrier, systematically tilting the immunological balance away from active surveillance toward sustained immune evasion (165, 166).

In addition to glucose, reprogramming of amino acid metabolism also plays a crucial role in immunomodulation within the TME, with alterations in the metabolism of glutamine, tryptophan, and arginine being particularly significant (168, 169). Glutamine is the most abundant free amino acid in plasma and tissue fluids and serves as a major carbon and nitrogen donor for tumor cells beyond glucose. Tumor cells often exhibit “addiction” to glutamine, upregulating glutaminase (GLS) to convert glutamine into glutamate, which is then metabolized to α-ketoglutarate to replenish the tricarboxylic acid (TCA) cycle-a process known as anaplerosis (170). The substantial glutamine consumption by tumor cells similarly limits the availability of substrates for T cells, thereby impairing their proliferation and differentiation (169, 170).

Dysregulation of tryptophan metabolism represents a major mechanism mediating immune tolerance mechanisms. Stimulated by inflammatory factors such as IFN-γ, tumor cells and APCs highly express IDO1 and tryptophan 2, 3-dioxygenase (TDO), which catalyze the metabolism of tryptophan along the kynurenine pathway. This process leads to local tryptophan depletion, not only triggering stress responses and cell cycle arrest in T cells through activation of the GCN2 kinase but also producing kynurenine, an endogenous ligand for the aryl hydrocarbon receptor (AhR). Kynurenine directly acts on T cells, promoting their differentiation into Tregs and thereby establishing an immunosuppressive microenvironment (171). Furthermore, arginine metabolism is primarily regulated by Arg-1, secreted by MDSCs and TAMs. Arg-1 hydrolyzes L-arginine into ornithine and urea, resulting in arginine deficiency in the microenvironment. Since the synthesis of the T cell CD3ζ chain is highly dependent on arginine, its depletion directly impairs the function of the TCR complex, thereby compromising antigen recognition and downstream signal transduction (172).

Lipid metabolic reprogramming, an emerging focus of recent research, highlights the impact of lipid supply-demand imbalance within the TME on cellular fate. Tumor cells typically exhibit enhanced *de novo* lipogenesis, upregulating key enzymes such as fatty acid synthase (FASN) and acetyl-CoA carboxylase (ACC) to meet the increased demand for biomembrane synthesis driven by rapid proliferation. This activation of lipid synthesis pathways is often driven by transcription factors such as the androgen receptor or sterol regulatory element-binding protein 1 (SREBP-1) (173). In contrast, immunosuppressive cell subsets within the TME, such as regulatory Tregs and M2-polarized macrophages, often rely preferentially on exogenous lipid uptake and FAO to sustain their survival and suppressive functions (174).

An abundance of lipids and oxidatively modified lipoproteins in the TME is frequently associated with metabolic dysfunction in DCs. Studies have found that lipid accumulation in the TME causes the buildup of truncated triglycerides within lipid droplets in DCs. This not only disrupts the assembly of the antigen-processing complex but also triggers ER stress and the unfolded protein response (UPR), impairing the ability of DCs to cross-present antigens and effectively activate CD8^+^ T cells. This phenomenon is referred to as “lipid-associated immune dysfunction” (175). Simultaneously, lipid metabolites secreted by tumor cells, such as prostaglandin E2 (PGE2), can directly suppress effector T cells and recruit MDSCs. Therefore, lipid metabolism serves not merely as a supplement to energy metabolism but also as a critical regulator of cellular signaling transduction and membrane fluidity, thereby substantially influencing the effector functions of immune cells (176).

The plasticity of mitochondrial function within the TME also has increasingly been recognized as a critical determinant of tumor adaptation. Despite the Warburg effect, mitochondria in tumor cells remain functionally active. Instead, they exhibit high plasticity and enable dynamic shifts between glycolysis and oxidative phosphorylation to adapt to varying microenvironmental stresses. For example, circulating tumor cells or dormant cancer stem cells often rely more heavily on mitochondrial oxidative phosphorylation (177). Furthermore, ROS generated by dysfunctional mitochondria can exert biphasic effects in the TME: moderate ROS levels promote oncogenic signaling, while excessive ROS induces cytotoxicity. Tumor cells maintain redox homeostasis by upregulating antioxidant defense systems, such as glutathione and NADPH pathways, whereas infiltrating lymphocytes frequently undergo apoptosis due to their inability to tolerate high ROS levels (178) (**Figure 4**).

**Figure 4:**
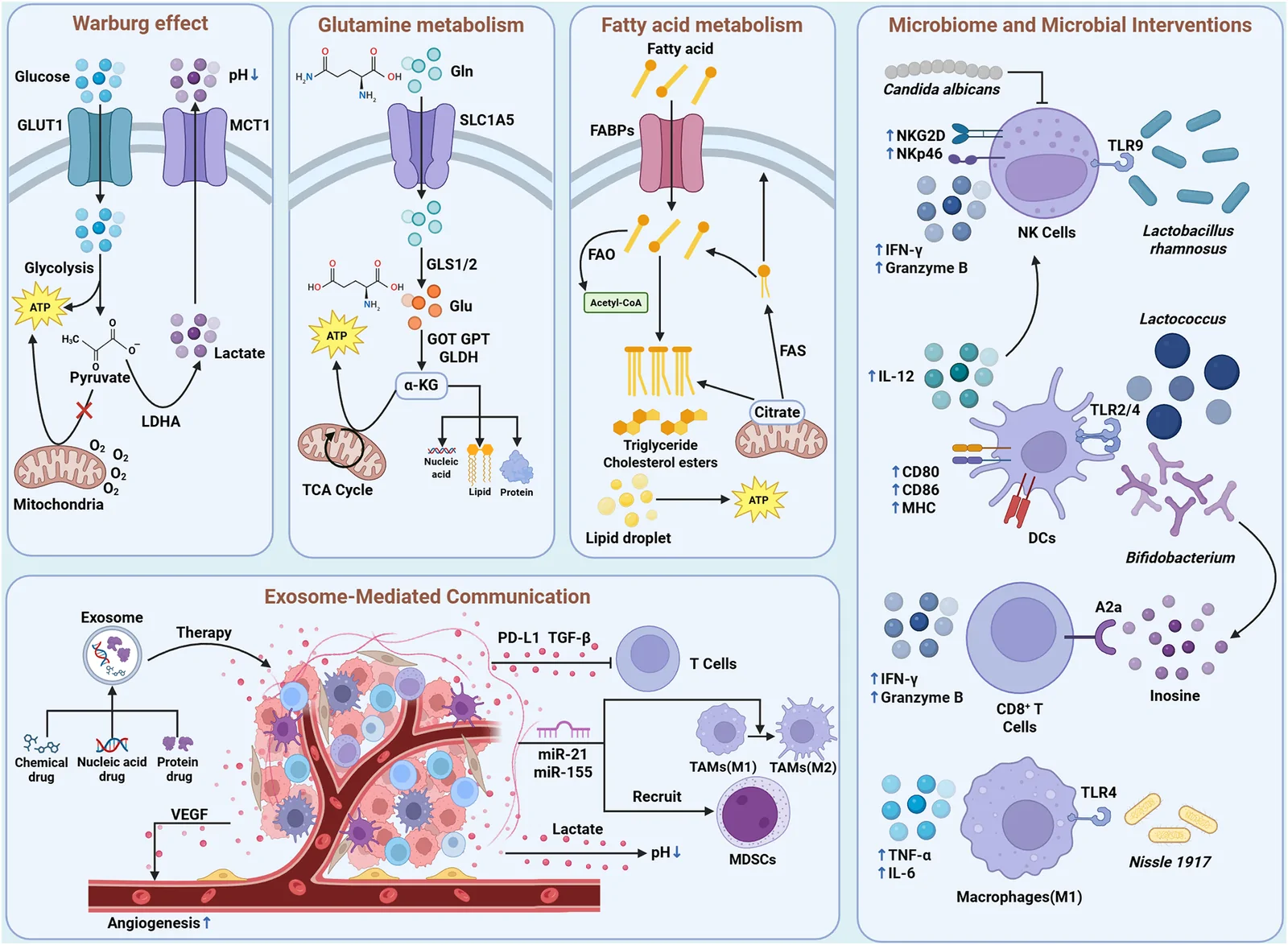
Metabolic reprogramming, microbial interventions, and exosome-mediated communication in the tumor microenvironment. This comprehensive schematic illustrates the complex intercellular regulatory networks within the TME. Tumor cells undergo significant metabolic adaptations, including the Warburg effect (aerobic glycolysis), enhanced glutamine metabolism to fuel the TCA cycle, and altered fatty acid metabolism. Concurrently, the TME is modulated by the microbiome, where specific probiotic strains (e.g., *Lactobacillus*, *Bifidobacterium*) and their metabolites (such as inosine) enhance anti-tumor immunity by activating dendritic cells, macrophages, and cytotoxic T and NK cells, whereas fungal pathogens like *Candida albicans* exert immunosuppressive effects. Furthermore, intercellular communication is heavily driven by tumor-derived exosomes, which deliver immunosuppressive molecules (e.g., PD-L1, TGF-β, miR-21/155) and metabolites (lactate) to inhibit T cell function, drive TAM polarization toward an M2 phenotype, recruit MDSCs, and promote angiogenesis. The diagram also highlights the promising therapeutic potential of engineered exosomes serving as targeted delivery vectors for chemical, nucleic acid, and protein drugs.

The metabolic remodeling within the TME encompasses mechanisms that transcend simple cellular bioenergetics. In essence, it represents an efficient “metabolic checkpoint” established by tumor cells. Whether through the competitive depletion of glucose and amino acids or the abnormal accumulation of lipids and lactate, these processes can collectively induce metabolic maladaptation and functional exhaustion in effector T cells. A deeper dissection of this intricate metabolic interaction network is essential for clarifying understanding how tumor cells reshape the immune microenvironment through metabolic dominance.

### 3.3 Extracellular vesicle mediated communication

Within the TME, intercellular communication is not only dependent on cytokine paracrine signaling and direct cell to cell contact but also involves a prominent class of extracellular vesicles known as EVs are nanometer-scale (approximately 30-150 nm in diameter) lipid bilayer-enclosed vesicles that originate from intracellular multivesicular bodies (MVBs) and are released into the extracellular space upon fusion with the plasma membrane. Unlike cellular debris or apoptotic bodies, the biogenesis and release of exosomes constitute a regulated process, involving coordinated actions of the endosomal sorting complex required for transport (ESCRT) and Rab GTPase family proteins (22). As vehicles for intercellular cargo and signal exchange between cells, EVs are widely present in the TME and are secreted not only by tumor cells but also by stromal and immune cells. Their unique lipid-bilayer structure confers high stability, protecting the enclosed bioactive molecules from degradation by extracellular enzymes and thereby enabling long-distance, trans-tissue signaling. This vesicle-based mode of communication is characterized by its diverse informational cargo, targeting specificity, and systemic functionality. As such, it has become a critical dimension for understanding tumor progression, immune evasion, and metastatic colonization (179).

The core of EVs mediated TME remodeling lies in their complex “cargo.” This cargo is not a random encapsulation of cellular components but is selectively enriched into vesicles through specific sorting mechanisms, encompassing proteins (such as receptors, enzymes, and transcription factors), lipids, and various nucleic acid molecules (including DNA, mRNA, miRNA, lncRNA, and circRNA). These bioactive molecules are taken up by recipient cells via receptor-mediated endocytosis, macropinocytosis, or membrane fusion. Once internalized, they are released into the cytoplasm, where they subsequently regulate gene expression at the post-transcriptional or translational level, thereby inducing phenotypic transformation (180). Within the TME, tumor-derived exosomes (TDEs) serve as the primary drivers of microenvironmental reshaping. By horizontally transferring oncogenic molecules, TDEs can functionally alter surrounding stromal cells and infiltrating immune cells, conditioning them toward pro-tumorigenic phenotypes that support tumor growth, thereby constructing a fertile soil conducive to tumor survival (181).

In terms of immune modulation, TDEs primarily exert immunosuppressive functions and represent a key mechanism underlying tumor immune evasion. TDEs carry a variety of immunosuppressive ligands or cytokines that act directly on effector T cells. For example, TDEs highly express FasL or TRAIL on their surface, which can bind to corresponding receptors on T cells and induce apoptosis in activated CD8^+^ T cells (10). Additionally, recent studies have confirmed that tumor cells can release PD-L1 protein via exosomes. This exosomal PD-L1 not only suppresses the priming and activation of T cells in distal lymph nodes but also function as a molecular sink in the circulation, neutralizing anti-PD-L1 therapeutic antibodies and thereby contributing to resistance against immune checkpoint blockade therapy (154, 155). Furthermore, specific miRNAs encapsulated within TDEs (such as miR-21 and miR-301a) can, upon uptake by macrophages, activate signaling pathways such as TLR/NF-κB or STAT3, driving macrophage polarization toward a pro-tumorigenic M2-like phenotype. These M2-like macrophages secrete IL-10 and TGF-β, further suppressing antitumor immune responses (182, 183). In addition, TDEs can impair the maturation of DCs and promote the expansion and recruitment of Tregs and MDSCs. Thus, TDEs help establish a comprehensive immunosuppressive network within the TME (184).

Besides remodeling the immune microenvironment, EVs also play a pivotal role in activating tumor stroma and promoting angiogenesis. Tumor cells secrete EVs containing TGF-β, SMADs, and specific lncRNAs, which act upon quiescent fibroblasts and drive their transdifferentiation into cancer-associated fibroblasts (CAFs). Activated CAFs subsequently synthesize large quantities of matrix components such as collagen and fibronectin, thereby remodeling the ECM and increasing tissue stiffness. Concurrently, they secrete MMPs to degrade the stromal barrier, establishing a physical niche permissive to tumor invasion (185). In the context of angiogenesis, tumor cells under hypoxic conditions secrete exosomes enriched with VEGF, HIF-1α, and pro-angiogenic miRNAs (e.g., miR-135b). Upon uptake by vascular endothelial cells, these EVs markedly enhance endothelial cell proliferation, migration, and tube-formation capacity, while also compromising vascular wall integrity and increasing vascular permeability. This promotes the formation of new tumor-associated blood vessels, thereby mitigating the localized hypoxia and nutrient deprivation that accompanies rapid tumor growth (186, 187).

EVs mediated communication plays a particularly prominent role in tumor metastasis, especially during the formation of the pre-metastatic niche (PMN). Primary tumor cells can release exosomes into the circulation, releasing EVs into the bloodstream that home to specific distant organs. Evidence indicates that the types of integrins present on the exosome surface largely dictate the organotropism of tumor metastasis, which refers to the organ-specific affinity. For example, EVs enriched with integrin α6β4 tend to target fibroblasts and epithelial cells in the lungs, whereas those rich in αvβ5 preferentially home to Kupffer cells in the liver (188). Upon reaching distant organs, these exosomes remodel the local microenvironment by inducing inflammatory responses, increasing vascular permeability, and recruiting bone marrow-derived suppressor cells. This process establishes a permissive niche for the survival and colonization of tumor cells, thereby conditioning the distant microenvironment before the actual arrival of tumor cells (189).

Notably, EVs mediated communication within the TME is not a one-way process dictated solely by tumor cells, but rather a bidirectional interplay. EVs derived from stromal and immune cells can also reciprocally modulate tumor cells, influencing their malignant phenotype. For instance, EVs secreted by CAFs have been demonstrated to deliver Wnt ligands or specific miRNAs to tumor cells, thereby activating the Wnt/β-catenin planar cell polarity pathway and inducing epithelial-mesenchymal transition (EMT). This confers increased invasiveness and stem-like properties to tumor cells (190). Moreover, stromal cells subjected to chemotherapy can transfer drug-efflux pump proteins or anti-apoptotic proteins to tumor cells via EVs, aiding tumor cells in acquiring chemoresistance (191) (**Figure 4**).

As key mediators of intercellular communication within the TME, EVs establish a multidimensional, bidirectional, and dynamic signaling regulatory network. This network transmits genetic information and protein signals to coordinate the interactions between tumor cells and host stromal or immune cells. It is widely implicated in driving in various pathological processes, including sustaining an immunosuppressive microenvironment, promoting angiogenesis, inducing therapy resistance, and driving distant metastasis. A systematic elucidation of the sorting mechanisms of exosomal cargo and the patterns of their transfer among specific cellular subsets remains of paramount importance. Such investigation will help clarify the spatiotemporal evolution of the tumor microenvironment. Moreover, it will provide a critical theoretical foundation for developing novel antitumor therapies, including strategies that block communication and approaches that employ EVs as drug delivery vehicles.

## 4 Therapeutic strategies targeting innate immunity

### 4.1 Reprogramming innate immune cells

Adoptive cell therapy has undergone decades of iterative innovation since the early milestone of allogeneic hematopoietic stem cell transplantation, which pioneered the modern cell therapy landscape and laid the foundation for immune cell-based tumor treatment. In its early developmental stage, the field predominantly focused on adaptive immune cells, with CAR-T cell therapy emerging as a transformative breakthrough and achieving remarkable clinical efficacy in hematological malignancies (192). Nevertheless, the inherent limitations of CAR-T cells, including the high risk of GVHD, severe cytokine release syndrome (CRS), neurotoxicity, stringent HLA matching requirements, and poor efficacy against solid tumors, have severely restricted its universal clinical application (193). Beyond engineered lymphocyte therapies, multiple immunomodulatory strategies targeting the tumor microenvironment are under active investigation: TREM2 and PI3Kγ inhibition reprograms immunosuppressive tumor-associated macrophages; blockade of NK cell checkpoints reinvigorates innate NK cell cytotoxicity; CXCR2 targeting curtails neutrophil recruitment and disrupts pro-tumor neutrophil extracellular traps (NETs); and dendritic cell activation potentiates antigen presentation to prime robust anti-tumor adaptive immunity. To overcome these bottlenecks and expand the therapeutic boundary of adoptive cell therapy, researchers have gradually shifted their research focus from adaptive lymphocytes to innate immune cells with unique biological advantages, driving a fundamental paradigm shift in cell reprogramming and tumor immunotherapy (104, 194) (**Table 2**).

**Table 2:** Key advances in cell therapy for oncology (2021–2025).

Therapy/Product	Preclinical evidence	Clinical evidence	Developmental stage	Indicated tumor types	Main limitations
Breyanzi (liso-cel, CD19 CAR-T)	Second-generation CAR with 4-1BB co-stimulatory domain. Preclinical studies demonstrated low T cell exhaustion and potent *in vitro* B-cell killing activity, supporting robust cell expansion at low doses.	FDA approved in 2021. Pivotal phase II TRANSCEND NHL 001 trial: ORR 73%, CR 54% in relapsed/refractory large B-cell lymphoma. MCL indication expanded in 2025.	Launched in USA (2021); indication expansion in 2025; parallel registration ongoing in China	Relapsed/refractory large B-cell lymphoma, mantle cell lymphoma	Autologous manufacturing with long production lead time; risk of CRS and ICANS; relapse driven by antigen loss; ineffective for CD19-negative malignancies; high cost
Abecma (idecabtagene vicleucel, BCMA CAR-T)	BCMA-specific CAR. Preclinical assays showed potent myeloma cell killing *in vitro* and marked tumor regression in animal models.	FDA approved in 2021. Phase II KarMMa trial: ORR 73%, CR 33% in relapsed/refractory multiple myeloma. Long-term follow-up confirmed OS benefit in patients with durable responses.	Launched in USA (2021); multiple domestic BCMA CAR-T products under registration in China	Relapsed/refractory multiple myeloma	BCMA downregulation or antigen loss; bone marrow microenvironment-mediated immune suppression; late-onset infections; hematopoietic injury; personalized autologous manufacturing
Carvykti (cilta-cabtagene autoleucel, BCMA CAR-T)	Dual-epitope BCMA CAR. Preclinical data showed higher antigen affinity and improved resistance to tumor escape compared with single-epitope BCMA CAR constructs.	FDA and NMPA approved in 2022. CARTITUDE-1 trial: ORR 97.9%, sCR 78%. Long-term follow-up demonstrated deep and sustained remissions.	Approved in USA and China (2022)	Relapsed/refractory multiple myeloma	Relatively high CRS rate; neurotoxicity; BCMA-negative relapse; myelosuppression; lengthy autologous cell production
Kimmtrak (tebentafusp, TCR-T)	gp100-targeted TCR with validated HLA restriction. Preclinical models demonstrated tumor growth inhibition in uveal melanoma.	FDA approved in 2022. Phase III trial in HLA-A*02:01-positive metastatic uveal melanoma: median OS 21.7 months vs 16 months in control arm.	Launched in USA (2022)	HLA-A*02:01-positive metastatic uveal melanoma	Strict HLA genotype restriction; TCR cross-recognition causing off-tumor toxicity; solid tumor microenvironment immune suppression; limited proportion of eligible patients
CT-0508 (HER2 CAR-M, Carisma Therapeutics)	Autologous monocyte-derived CAR macrophages targeting HER2. Preclinical data showed tumor phagocytosis, secretion of pro-inflammatory cytokines (IL-12, TNF-α), TME remodeling and recruitment of CD8^+^ T cells, with robust tumor control in mouse solid tumor models.	World’s first CAR-M phase I trial (NCT04660929). 14 patients with HER2-overexpressing advanced solid tumors. 44% (4/9) of HER2 IHC 3+ patients achieved stable disease; all HER2 IHC 2+ patients progressed. No DLT or grade ≥3 CRS/ICANS; grade 1–2 CRS (64.3%) resolved within 1–4 days. Manufacturing success rate 100%. One heavily pretreated inflammatory breast cancer case showed 93% ctDNA reduction.	Phase I clinical trial; ongoing combination substudy with pembrolizumab	HER2-overexpressing advanced solid tumors (breast, gastroesophageal, salivary gland carcinoma)	Limited efficacy (only SD observed, no RECIST objective responses); efficacy strongly dependent on high HER2 expression; short *in vivo* persistence; autologous manufacturing; TME immunosuppression remains a major barrier
Tecelra (afami-cel, NY-ESO-1 TCR-T)	NY-ESO-1-targeted affinity-optimized TCR. Preclinical studies achieved potent tumor suppression in synovial sarcoma models.	FDA accelerated approval in 2024. SPEARHEAD-1 phase II trial: ORR 51% in synovial sarcoma.	Approved in USA (2024)	Advanced synovial sarcoma, myxoid round cell liposarcoma	Reliance on NY-ESO-1 antigen expression; HLA restriction; TME immune suppression; risk of off-tumor reactivity
Lifileucel (TIL)	Ex vivo expanded tumor-infiltrating lymphocytes. Preclinical validation of neoantigen recognition and tumor control in mouse solid tumor models.	FDA approved in 2024. Registration phase II trial in unresectable metastatic melanoma: ORR 31.4%, with several patients achieving durable CR. The world’s first commercial TIL product.	Launched in USA (2024); domestic TIL pipeline (GC101) in phase II	Advanced melanoma; clinical expansion to cervical cancer, NSCLC and other solid tumors	Tumor biopsy requirement; high expansion failure rate; mandatory lymphodepletion plus high-dose IL-2; severe systemic toxicity from IL-2; long manufacturing timeline
RR-M01/BM-1901 (HER2 CAR-M, Kunshi Biotech)	Third-generation HER2 CAR-M combined with gene editing and biomaterial engineering. Preclinical data showed improved *in vivo* survival and anti-tumor potency in prostate and pancreatic cancer models.	First-in-human administration of third-generation CAR-M completed in 2024 (IIT, Xuzhou Medical University Affiliated Hospital). Enrollment ongoing; 2 DS-8201-refractory HER2 IHC 3+ patients achieved PR; DCR ~71.4%. Updated data reported at ESMO 2025; IND filing initiated in China and USA in 2025.	Investigator-initiated trial; IND application ongoing	HER2-positive/low-expression solid tumors; pancreatic cancer, small cell lung cancer, ovarian cancer, peritoneal mesothelioma	Small IIT cohort without confirmatory registration trial; long-term safety and durability remain unvalidated; autologous cell preparation; limited cross-tumor clinical data
Off-the-shelf iPSC CAR-NK	iPSC-derived NK cells engineered with CAR and edited inhibitory receptors. Preclinical proof of off-the-shelf allogeneic product with low rejection risk; efficacy demonstrated in hematologic malignancies and select solid tumor mouse models.	Multiple phase I/II studies for CD19 CAR-NK in relapsed/refractory B-cell malignancies. Markedly lower CRS and ICANS compared with CAR-T. Phase I solid tumor trials ongoing.	Phase I/II clinical trial	Relapsed/refractory B-cell lymphoma, AML; early exploration in liver cancer, ovarian cancer	Short *in vivo* persistence; poor solid tumor infiltration; potential oncogenic risk from gene editing; challenges in process scaling
Solid-tumor targeted CAR-T (GPC3/MSLN/HER2)	Multiple CAR constructs targeting GPC3, MSLN etc. Preclinical mouse models showed tumor suppression in hepatocellular carcinoma, mesothelioma. Logic-gated CAR designs reduce on-target off-tumor toxicity.	Phase I/II trials with generally low ORR. Partial tumor shrinkage observed in subsets of GPC3 CAR-T liver cancer patients, yet durable responses are rare; TME is the primary bottleneck.	Phase I/II clinical trial	Hepatocellular carcinoma, mesothelioma, ovarian cancer, HER2-positive solid tumors	Immunosuppressive solid tumor microenvironment; tumor antigen heterogeneity; on-target off-tumor toxicity; poor tumor infiltration; antigen escape
*In vivo* CAR-T (LNP-mRNA)	LNP-encapsulated mRNA for *in situ* T cell reprogramming. Preclinical mouse hematologic malignancy models demonstrated anti-tumor activity without ex vivo cell manufacturing.	Early phase I for *in vivo* CD19 CAR-T; preliminary safety confirmed; efficacy remains to be established.	Phase I clinical trial	B-cell malignancies (hematologic tumors prioritized)	Low *in vivo* transfection efficiency; targeted delivery hurdles; off-target gene editing risk; insufficient *in vivo* persistence

The paradigm of immune cell reprogramming is now undergoing a significant evolution, expanding beyond the constraints of adaptive lymphocytes to harness the unique biological properties of the innate immune compartment. This shift is primarily driven by the imperative to develop broadly applicable, “off-the-shelf” allogeneic therapies that mitigate the risk of GVHD while offering distinct mechanisms of tumor infiltration and immune modulation (195). NK cells, as the key cytotoxic effectors of the innate immune system, have emerged as promising candidates for CAR engineering. CAR-NK cells offer a compelling safety profile; unlike T cells, allogeneic NK cells are generally not associated with inducing GVHD, potentially circumvents the need for rigorous HLA matching or complex gene-editing strategies to knock out endogenous T-cell receptors. Furthermore, the physiological lifespan of NK cells is relatively short, which may help limit the duration of potential adverse events, and their distinct cytokine secretion profile-predominantly producing IFN-γ and GM-CSF rather than IL-6-significantly lower the incidence and severity of CRS and neurotoxicity (196). Beyond their engineered CAR specificity, CAR-NK cells retain their intrinsic, CAR-independent mechanisms of tumor recognition. They possess a repertoire of germline-encoded activating and inhibitory receptors, governed by the “missing-self” principle, which allows them to recognize and eliminate tumor cells that downregulate HLA class I molecules-a common evasion strategy utilized against TCR-T and endogenous CD8^+^ T cells (42). Additionally, NK cells can exert cytotoxicity via ADCC through the CD16 receptor, providing a mechanistic rationale for combinatorial approaches for combinatorial therapies with monoclonal antibodies (197). To optimize the efficacy of CAR-NK cells, contemporary engineering strategies often incorporate NK-specific signaling domains, such as DAP10, DAP12, or 2B4 (CD244), to better recapitulate natural NK activation pathways, alongside the integration of transgenic cytokines like IL-15 to enhance *in vivo* persistence and expansion (198). Despite their therapeutic potential, the clinical deployment of CAR-NK cells is challenged by their relative resistance to viral transduction, the technical complexities of efficient cryopreservation without compromising viability or cytotoxic function, and the necessity to sustain their persistence in the highly immunosuppressive microenvironment of solid tumors (43).

Addressing the substantial physical and immunological barriers of solid tumors has precipitated the development of Chimeric Antigen Receptor Macrophages (CAR-M). Macrophages are widely distributed throughout human tissues and possess an intrinsic capacity, to infiltrate solid tumors, primarily driven by chemotactic gradients such as CCL2 secreted by the tumor stroma. Within the TME, TAMs are frequently co-opted by the TME into a pro-tumorigenic M2-like pro-tumorigenic phenotype, promoting angiogenesis, immunosuppression, and metastasis (199). However, CAR engineering effectively reprograms these innate phagocytes, not only directing their phagocytic activity against specific tumor antigens but also simultaneously driving a phenotypic shift toward a pro-inflammatory, anti-tumor M1-like state. The CAR-M construct typically utilizes the CD3ζ intracellular domain or Megf10/FcRγ pathways to trigger actin remodeling and target engulfment (200, 201). Upon encountering the target antigen, CAR-M cells execute targeted phagocytosis, actively degrading the malignant cells. Importantly, this process extends beyond direct cytotoxicity. As professional APCs, CAR-M cells process the engulfed tumor material and present tumor-derived antigens to surrounding T cells. This mechanism effectively bridges the innate and adaptive immune responses, triggering epitope spreading and initiating a broader, durable endogenous anti-tumor immune response capable of targeting antigen-loss variants. Furthermore, the localized secretion of pro-inflammatory cytokines and chemokines by engineered macrophages significantly remodels the surrounding TME, recruiting endogenous effector T cells and NK cells while mitigating the suppressive influence of Tregs and TAMs. While CAR-M therapy represents a promising strategy to solid tumors, it is not without substantial obstacles. Primary human macrophages are notably refractory to standard lentiviral or retroviral transduction, often necessitating the use of adenoviral vectors, chimeric viral platforms, or advanced non-viral delivery methods such as lipid nanoparticles to achieve adequate engineering efficiency (202). Additionally, their limited ex vivo proliferation capacity has driven interest in the exploration of induced pluripotent stem cell (iPSC)-derived macrophages to generate sufficient cell yields for clinical application (203). Ultimately, TME-modulating capabilities of CAR-NK and CAR-M cells illustrates a comprehensive diversification of the adoptive cell therapy therapeutic landscape. By leveraging the distinct and synergistic physiological mechanisms inherent to both the adaptive and innate branches of the immune system, the field of cellular reprogramming is establishing multifaceted strategies designed to address the profound heterogeneities and evolutionary evasion mechanisms characteristic of refractory malignancies (204, 205) (**Figure 5**).

**Figure 5:**
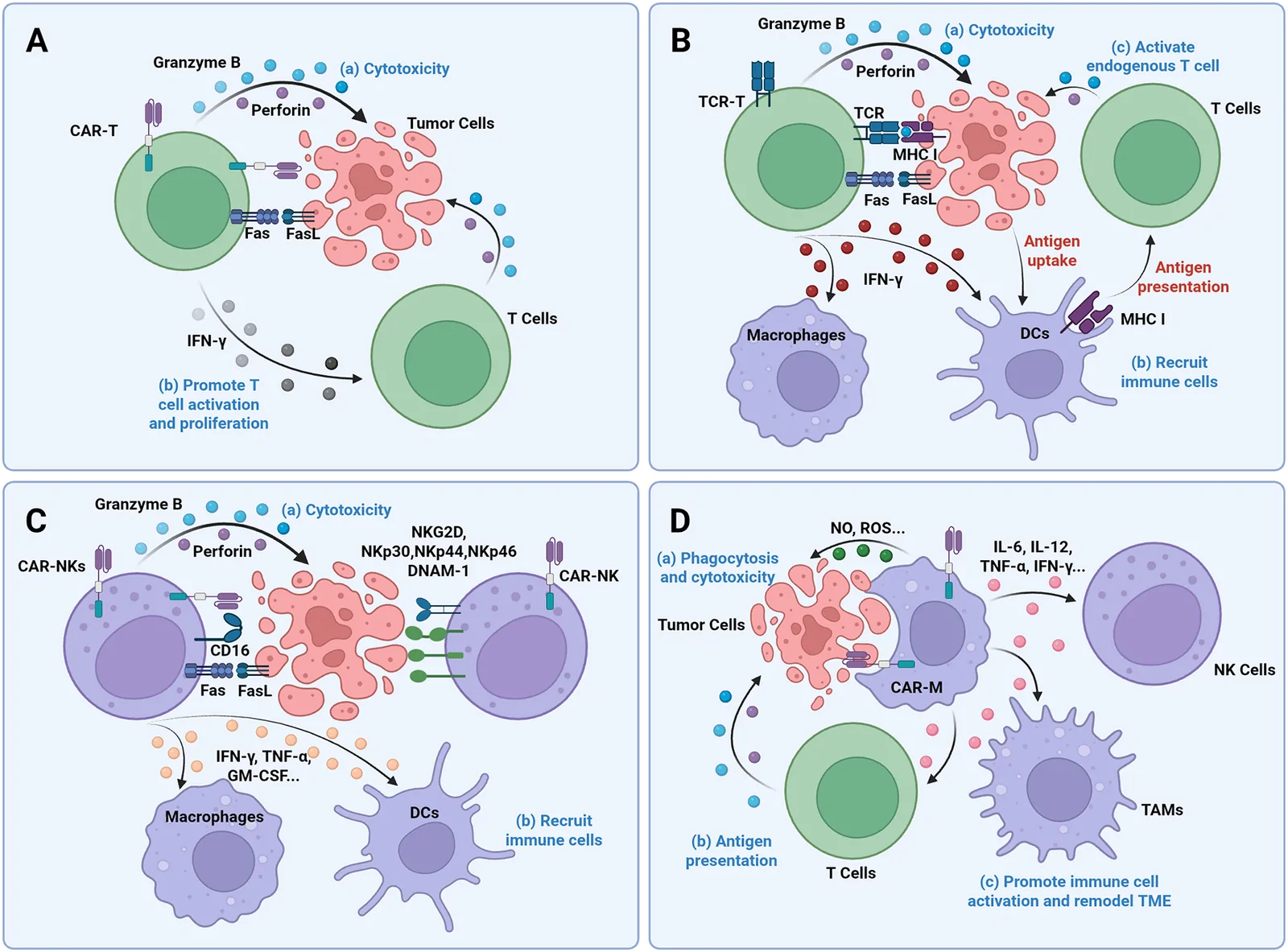
Mechanisms of action for engineered adoptive cell therapies in the tumor microenvironment. This schematic delineates the diverse, multi-faceted anti-tumor mechanisms of four distinct types of engineered immune cells. **(A)** CAR-T Cells induce direct cytotoxicity against tumor cells via the release of Granzyme B and Perforin, alongside Fas/FasL-mediated apoptosis. They also secrete IFN-γ to promote the activation and proliferation of endogenous T cells. **(B)** TCR-T Cells exert direct cytotoxic effects and secrete cytokines (e.g., IFN-γ) to recruit macrophages and dendritic cells (DCs). These DCs subsequently uptake tumor antigens and cross-present them via MHC I to further activate endogenous T cells. **(C)** CAR-NK Cells mediate potent cytotoxicity through CAR engagement, CD16-mediated ADCC, and innate activating receptors (NKG2D, NKp30/40/46, DNAM-1). Furthermore, they secrete cytokines like IFN-γ, TNF-α, and GM-CSF to recruit and activate other innate immune cells. **(D)** CAR-M (Macrophages) eliminate tumor cells through direct phagocytosis and the release of cytotoxic molecules (NO, ROS). Crucially, CAR-Ms also function as professional antigen-presenting cells to prime T cells and secrete an array of pro-inflammatory cytokines (IL-6, IL-12, TNF-α, IFN-γ) to systemically activate NK cells and remodel the immunosuppressive TME.

### 4.2 Engineered microbial therapies

Harnessing the intrinsic biological properties of microbes to modulate the innate immune response within the TME has emerged as a promising therapeutic strategy. Recent advances in synthetic biology and genetic engineering have enabled the rational design of engineered microbes, particularly reprogrammed bacteria and oncolytic viruses, to counteract the immunosuppressive TME and restore the anti-tumor functions of innate immune cells (206).

Many anaerobic or facultatively anaerobic bacteria, such as *Escherichia coli* (*E. coli*), possess intrinsic tropism toward the hypoxic and necrotic regions characteristic of solid tumors. This intrinsic tumor-targeting colonization capability makes them suitable chassis for anti-tumor drug delivery (207). Using synthetic biology approaches, E. coli can be engineered, to achieve the spatiotemporally controlled release of immunomodulatory payloads within the TME (208), thereby remodeling the innate immune microenvironment (209).

For instance, researchers have engineered attenuated *E. coli* strains equipped with synchronized lysis circuits. Once the bacterial population reaches a threshold population density within the tumor, it triggers self-lysis, releasing localized therapeutic immunomodulators (208). Furthermore, engineered *E. coli* can be engineered to secrete nanobodies that block immune checkpoints, such as anti-CD47 nanobodies. This intervention not only disrupts the CD47-mediated anti-phagocytic signal on tumor cells but also directly promotes phagocytosis by macrophages, driving the polarization of TAMs from a pro-tumorigenic M2 phenotype to an anti-tumorigenic M1 phenotype. By continuously stimulating pattern recognition receptors (PRRs) on innate immune cells, these reprogrammed bacteria help establish a localized inflammatory microenvironment that facilitates DC maturation and antigen presentation.

Oncolytic viruses represent another valuable modality of engineered microbial therapy. Their key advantage lies in their capacity to selectively infect and lyse malignant cells while concomitantly eliciting a substantial innate immune response. The anti-tumor mechanism of OVs is multifaceted, primarily driven by direct oncolysis followed by the induction of ICD. Viral-mediated lysis results in the widespread release of TAAs, damage-associated molecular patterns (DAMPs, such as ATP and HMGB1), and pathogen-associated molecular patterns (PAMPs, including viral nucleic acids) directly into the TME (210).

These released DAMPs and PAMPs are rapidly recognized by innate immune sensors-including TLRs, RIG-I-like receptors (RLRs), and the cGAS-STING pathway-thereby triggering a Type I interferon response in immunosuppressive “cold” tumors (211). To further amplify this cascade, OVs are frequently reprogrammed to encode innate immune-stimulating factors. For example, engineered viruses expressing GM-CSF significantly enhance the recruitment and activation of DCs, effectively bridging innate immunity with adaptive T-cell responses (212). Next-generation OVs are additionally being engineered to express cytokines such as IL-12 or chemokines such as CCL5, which augment the infiltration and cytotoxicity of NK cells (213) (**Figure 6**).

**Figure 6:**
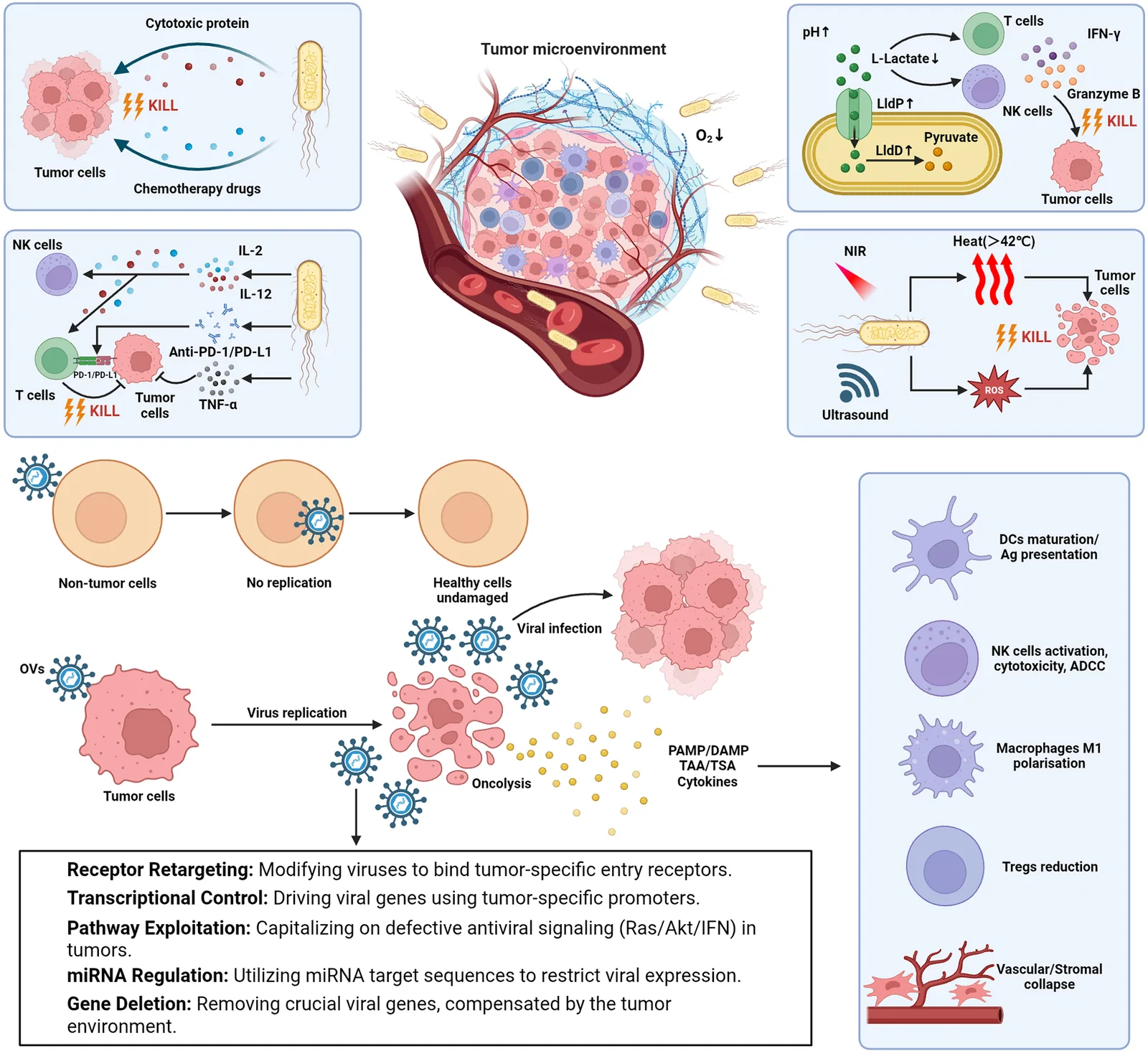
Therapeutic mechanisms of engineered bacteria and oncolytic viruses in the tumor microenvironment. This schematic illustrates the multifaceted modalities of microbe-based tumor therapies. Bacterial Therapies: Engineered bacteria selectively colonize the hypoxic tumor microenvironment to exert multi-pronged anti-tumor effects. They can directly deliver cytotoxic proteins and chemotherapeutic drugs, synergize with immune checkpoint blockade (anti-PD-1/PD-L1) by secreting immunostimulatory cytokines (e.g., IL-2, IL-12), and remodel the acidic TME by consuming L-lactate to restore T cell and NK cell cytotoxicity. Additionally, these bacteria can mediate photothermal and sonodynamic therapies (triggered by NIR and ultrasound, respectively) to induce tumor cell death via hyperthermia and ROS generation. OV Therapies: OVs are rationally designed via multiple engineering strategies (receptor retargeting, transcriptional control, pathway exploitation, miRNA regulation, and gene deletion) to selectively infect and replicate within tumor cells while sparing healthy tissue. Viral replication culminates in tumor cell lysis, releasing PAMPs, DAMPs, TAA, and cytokines. This immunogenic cascade robustly activates anti-tumor immunity, driving DC maturation, NK cell activation, macrophage M1 polarization, Treg depletion, and the collapse of tumor vasculature and stroma.

### 4.3 Combination therapies

Although monotherapies targeting innate immune pathways have shown promising results in preclinical models, their clinical efficacy is often constrained by the complex compensatory mechanisms and redundant immunosuppressive networks within the TME. A growing pipeline of novel innate-targeted cancer agents has entered early-phase clinical trials over recent years yet still fails to overcome single-agent resistance in most patients. Therefore, rationally combining innate immune modulators with the standard of care or other immunotherapies has become an important approach to mitigate immune tolerance and improve clinical response rates (214) (**Table 3**).

**Table 3:** Key advances in oncology drug clinical research (2021–2025).

Drug/Agent	Preclinical evidence	Clinical evidence	Developmental stage	Indicated tumor types	Main limitations
Sotorasib (Lumakras, KRAS G12C small-molecule inhibitor)	Covalently binds KRAS G12C and locks the protein in GDP-bound inactive conformation. *In vitro* and xenograft models demonstrate robust suppression of KRAS G12C tumor proliferation with minimal activity against wild-type KRAS.	FDA accelerated approval in 2021. Phase II CodeBreaK100: ORR 37.1%, mPFS 6.8 months, mOS 12.5 months for pretreated KRAS G12C NSCLC. Phase III CodeBreaK200 confirmed superiority over docetaxel.	Launched in USA (2021); approved in China (2022)	Metastatic KRAS G12C-mutant non-small cell lung cancer	Acquired resistance via secondary KRAS mutations and bypass signaling; limited activity in brain metastases; restricted to one mutation subtype; gastrointestinal toxicity; non-curative
Trastuzumab deruxtecan (DS-8201, Enhertu, HER2 ADC)	High DAR (~8) ADC with topoisomerase I payload; potent bystander killing. *In vitro* and PDX models show strong cytotoxicity even in HER2-low tumors.	FDA approval expanded in 2022 for HER2-mutant NSCLC (DESTINY-Lung01 ORR 54.9%, mPFS 8.2 m, mOS 18.6 m). Phase III DESTINY-Breast04 (2023) established survival benefit in HER2-low breast cancer.	US approval 2022/2023; China approval 2023	HER2-positive/HER2-low breast cancer; HER2-mutant NSCLC; HER2-positive gastroesophageal cancer	Risk of interstitial lung disease; myelosuppression; tumor antigen heterogeneity; resistance via HER2 downregulation or payload tolerance; infusion-related reactions
Relatlimab + Nivolumab (Opdualag, LAG-3/PD-1 fixed combination)	Dual blockade of LAG-3 and PD-1 reverses T-cell exhaustion. Single-agent activity modest in tumor-bearing mice; combination restores CD8^+^ T-cell function.	FDA approval in 2022. Phase II/III RELATIVITY-047: unresectable melanoma mPFS 10.1 months vs 4.6 months for nivolumab monotherapy; durable OS benefit.	Launched in USA (2022)	Unresectable or metastatic melanoma	Increased cumulative immune-related adverse events; efficacy correlates with LAG-3 expression; weak single-agent activity in most solid tumors; high cost; only subset achieves durable remission
Mirvetuximab soravtansine (Elahere, FRα ADC)	Folate receptor α-targeted ADC with maytansinoid payload; potent killing in FRα-high ovarian cancer PDX models with bystander cytotoxicity.	FDA accelerated approval in 2022. Phase II SORAYA: ORR 42%, mDOR 6.3 months in platinum-resistant ovarian cancer.	Launched in USA (2022)	FRα-positive platinum-resistant epithelial ovarian, fallopian tube and primary peritoneal carcinoma	Ocular toxicity (keratopathy); diarrhea and myelosuppression; efficacy highly dependent on FRα expression; frequent secondary resistance; limited to FRα-positive patients
Adagrasib (Krazati, KRAS G12C small-molecule inhibitor)	Irreversible KRAS G12C inhibitor with additional SHP2 inhibition; superior potency in preclinical models and blood-brain barrier penetration.	FDA accelerated approval in 2022. Phase II KRYSTAL-1: ORR 43%, mOS 12.6 months in KRAS G12C NSCLC; intracranial ORR 33% for brain metastases.	Launched in USA (2022); phase III ongoing in China	Metastatic KRAS G12C-mutant NSCLC; colorectal cancer in clinical expansion	Severe GI toxicities (nausea/vomiting/diarrhea); QT prolongation; acquired resistance; inability to eradicate minimal residual disease; limited durability of intracranial responses
Tarlatamab (Zepzelca,DLL3 × CD3 bispecific antibody)	DLL3/CD3 T-cell engager; redirects T cells to kill DLL3-positive SCLC *in vitro* and suppresses tumor growth in mouse models.	FDA accelerated approval in 2024. Phase II: ORR 40%, mOS 11.4 months in platinum-refractory extensive-stage SCLC.	Launched in USA (2024)	Platinum-refractory extensive-stage small cell lung cancer	CRS; anemia and thrombocytopenia; DLL3 antigen heterogeneity; lack of validated predictive biomarker; high relapse rate
Datopotamab deruxtecan (Dato-DXd, Datroway, Trop2 ADC)	Trop2-targeted ADC with topoisomerase I payload, high DAR; broad anti-tumor activity across Trop2-positive PDX models with bystander killing.	FDA approval in 2025. Phase III TROPION-Breast01 demonstrated superior PFS versus chemotherapy for HR+/HER2− advanced breast cancer.	Launched in USA (2025)	HR-positive HER2-negative metastatic breast cancer; clinical expansion into NSCLC	Interstitial lung disease risk; gastrointestinal toxicity; Trop2 expression heterogeneity; late-line resistance; long-term real-world tolerability under investigation
Telisotuzumab vedotin (Teliso-V, c-MET ADC)	c-MET antibody conjugated to MMAE payload; selective cytotoxicity in c-MET-overexpressing tumor cells and MET-altered NSCLC PDX models.	FDA approval in 2025. Phase II LUMINOSITY: ORR 51% in c-MET IHC3+ advanced non-squamous NSCLC.	Launched in USA (2025)	c-MET overexpressed advanced non-squamous NSCLC	Efficacy strictly dependent on high c-MET protein expression; peripheral neuropathy; gastrointestinal toxicity; bypass resistance in MET pathway; assay standardization challenges
ARV-471 (PROTAC, ER degrader)	ER-targeted PROTAC induces proteasomal degradation of ERα; suppresses ER+ breast cancer PDX growth and overcomes endocrine resistance.	Phase I proof-of-concept shows dose-dependent ER degradation; mostly SD with rare PR; no pivotal phase II/III registration data.	Phase I clinical trial	ER+/HER2− advanced breast cancer	Suboptimal oral bioavailability; risk of off-target protein degradation; unknown long-term safety; undefined predictive biomarkers; solid-tumor delivery barriers

The clinical success of ICIs, such as anti-PD-1/PD-L1 and anti-CTLA-4 antibodies, depends largely on the preexisting infiltration and activation of tumor-infiltrating lymphocytes (TILs). However, a significant proportion of patients present with “cold” tumors, which lack sufficient DC antigen presentation and pro-inflammatory cytokine support, leading to primary resistance to ICIs (215). Combining ICIs with innate immune stimulators-such as cGAS-STING agonists, TLR agonists, or oncolytic viruses-can induce Type I interferon production. This innate immune activation facilitates DC maturation, enhances antigen cross-presentation, and promotes the secretion of key chemokines such as CXCL9 and CXCL10. Consequently, this cascade recruits and primes CD8^+^ T cells, effectively converting “cold” tumors into “hot” inflamed tumors and reversing ICI resistance (6, 216). Additionally, combining CD47-SIRPα blockade with ICIs not only promotes macrophage-mediated phagocytosis but also amplifies downstream T-cell responses through enhanced antigen processing, achieving a functional synergy between innate and adaptive immunity (217).

Conventional treatments, including radiotherapy and specific chemotherapeutic agents (e.g., doxorubicin and oxaliplatin), do more than directly kill tumor cells; they can also induce ICD. ICD is characterized by the release of DAMPs and TAAs into the TME. However, without concurrent innate immune amplification, the immunostimulatory effects of these endogenous adjuvants are often rapidly suppressed by the TME (218). By integrating radiotherapy or chemotherapy with innate immune-targeting agents-such as macrophage repolarizing agents or NK cell activators-a synergistic matrix is formed. The cytotoxic therapies act as an *in-situ* vaccine by releasing antigens, while the innate immunomodulators (e.g., CSF-1R inhibitors targeting M2 TAMs) clear immunosuppressive hurdles. This multimodal approach not only enhances local tumor control but can also trigger an abscopal effect, leading to the systemic responses against distant metastatic lesions (219).

Anti-angiogenic therapies, primarily targeting the VEGF/VEGFR signaling pathway, can transiently induce tumor vascular normalization, thereby alleviating TME hypoxia and elevated interstitial fluid pressure (220). Because hypoxia acts as a major driver for the recruitment and polarization of immunosuppressive cells such asM2-like TAMs and MDSCs, alleviating it substantially alters the innate immune landscape. When anti-angiogenic agents or specific oncogenic pathway inhibitors (e.g., MAPK inhibitors) are combined with innate immune modulators (such as STAT3 inhibitors or myeloid checkpoint blockades), the combination dismantles the TME’s suppressive shield from both structural and molecular dimensions. This dual-pronged strategy effectively rescues NK cells and macrophages from functional dysfunction, restoring their innate immune surveillance and tumoricidal capacities (221).

In summary, combination therapies represent extend beyond simple additive effects. leveraging the mechanistic complementarity between innate and adaptive immune cell populations. Through spatiotemporally coordinated and multi-targeted interventions, rationally designed combination regimens aim to overcome TME barriers, offering deeper and more durable clinical responses against heterogeneous and refractory malignancies (222) (**Figure 6**).

## 5 Prospect

Innate immunity-centered tumor immunotherapy has achieved remarkable breakthroughs, yet multiple bottlenecks remain to be resolved for broader clinical translation (223). First, the high phenotypic plasticity of innate immune cells constitutes a core obstacle. TAMs, TANs, ILCs and γδ T cells readily shift toward immunosuppressive states under the metabolic and inflammatory pressure of the TME (224). Future research needs to decode the epigenetic and metabolic molecular switches governing their polarization and develop precise small-molecule modulators or gene-editing tools to stably lock innate cells into anti-tumor phenotypes, avoiding transient therapeutic effects caused by phenotype reversion (223, 225).

The heterogeneity of solid tumors limits the efficacy of single-target innate immune interventions. Current strategies targeting PD-1/PD-L1, cGAS-STING or single innate subsets fail to remodel cold tumors comprehensively (226). Multimodal combination regimens will become the mainstream direction: pairing CAR-NK/CAR-M adoptive cell therapy with STING agonists, engineered oncolytic microbes or metabolic regulators can simultaneously relieve immune suppression, boost antigen presentation and normalize tumor vasculature, converting immune-excluded tumors into inflamed hot tumors (227, 228). In addition, exosome-targeted interventions deserve deeper exploration; blocking immunosuppressive tumor-derived exosomes or engineering therapeutic exosomes to deliver cytokines and checkpoint inhibitors will provide minimally invasive liquid therapeutic approaches (229–231).

Translational gaps between basic research and clinical practice require bridging. Most preclinical results rely on immunocompetent mouse models, which cannot fully recapitulate human TME characteristics, microbiome composition and innate cell heterogeneity (232, 233). Single-cell spatial transcriptomics and multi-omics technologies should be applied to map human innate immune landscapes across diverse malignancies, identifying subtype-specific predictive biomarkers to screen patients who benefit from innate immunity therapy (234). Moreover, off-the-shelf allogeneic innate cell products, especially CAR-NK and CAR-γδ T cells, need optimized production, cryopreservation and safety control systems to lower manufacturing costs and eliminate GVHD risks (235, 236).

Finally, safety management demands further optimization. Overactivation of innate signaling pathways may trigger systemic inflammatory response syndrome, while long-term innate immune remodeling may disrupt tissue immune homeostasis. Balancing anti-tumor potency and autoimmune risk via spatiotemporal controllable gene circuits and tumor-specific delivery carriers will lay a solid foundation for safe, precise innate immunotherapy. Overall, unraveling the dual regulatory mechanisms of innate immune cells and developing multi-target combinatorial regimens will unlock the full potential of innate immunity, bringing durable curative benefits to refractory cancer patients (237).
